# Interaction Between the PERK/ATF4 Branch of the Endoplasmic Reticulum Stress and Mitochondrial One-Carbon Metabolism Regulates Neuronal Survival After Intracerebral Hemorrhage

**DOI:** 10.7150/ijbs.93787

**Published:** 2024-08-06

**Authors:** Yikui Liu, Fengzhen Cui, Aoqian Xu, Baofeng Wang, Yuxiao Ma, Qixiang Zhang, Qingfang Sun, Yongtao Zheng, Yuxiao Xue, Yuhao Sun, Liuguan Bian

**Affiliations:** 1 Department of Neurosurgery, Ruijin Hospital, Shanghai Jiao Tong University School of Medicine, Shanghai, China.; 2 Med-X Research Institute and School of Biomedical Engineering, Shanghai Jiao Tong University, Shanghai, China.; 3 School of Public Health, Guangdong Medical University, Dongguan, China.

**Keywords:** intracerebral hemorrhage, endoplasmic reticulum stress, mitochondria, one-carbon metabolism, GRP78/ATF4/MTHFD2

## Abstract

Recent investigations have revealed that oxidative stress can lead to neuronal damage and disrupt mitochondrial and endoplasmic reticulum functions after intracerebral hemorrhage (ICH). However, there is limited evidence elucidating their role in maintaining neuronal homeostasis. Metabolomics analysis, RNA sequencing, and CUT&Tag-seq were performed to investigate the mechanism underlying the interaction between the PERK/ATF4 branch of the endoplasmic reticulum stress (ERS) and mitochondrial one-carbon (1C) metabolism during neuronal resistance to oxidative stress. The association between mitochondrial 1C metabolism and the PERK/ATF4 branch of the ERS after ICH was investigated using transcription factor motif analysis and co-immunoprecipitation. The findings revealed interactions between the GRP78/PERK/ATF4 and mitochondrial 1C metabolism, which are important in preserving neuronal homeostasis after ICH. ATF4 is an upstream transcription factor that directly regulates the expression of 1C metabolism genes. Additionally, the GRP78/PERK/ATF4 forms a negative regulatory loop with MTHFD2 because of the interaction between GRP78 and MTHFD2. This study presents evidence of disrupted 1C metabolism and the occurrence of ERS in neurons post-ICH. Supplementing exogenous NADPH or interfering with the PERK/ATF4 could reduce symptoms related to neuronal injuries, suggesting new therapeutic prospects for ICH.

## Introduction

Stroke is a significant social and public health challenge that severely threatens human well-being and life, with a reported incidence of 2.8% among adults 1. While hemorrhagic stroke accounts for only approximately 10-37.6% of all stroke cases, it has a high morbidity (i.e., various neurological impairments) and an alarmingly high mortality rate of 50% for intracerebral hemorrhage (ICH) within 30 days of onset 1-4. Following ICH onset, oxidative stress precipitates a substantial consumption of antioxidants and accumulation of oxidants within neurons, disbalancing the oxidative and antioxidative processes and ultimately leading to neuronal apoptosis 5. Thus, inhibiting neuronal damage caused by oxidative stress is pivotal in treating ICH 5.

Highly active electrophysiological processes occur in neurons, requiring substantial energy to maintain homeostasis and normal physiological functions 6. Mitochondria act as a primary energy source for neurons through its electron transport and oxidative phosphorylation processes. However, mitochondria also generate large amounts of reactive oxygen species (ROS), which can trigger protein and lipid peroxidation, resulting in DNA damage and subsequent apoptosis 6-8. Nonetheless, cells possess an intrinsic antioxidant system that maintains ROS levels in equilibrium and manages excess ROS during pathological situations 7. Nicotinamide Adenine Dinucleotide Phosphate Hydrogen (NADPH) plays a vital role in supplying electrons to antioxidant systems and supporting biosynthesis through reduction processes, which are crucial for counteracting oxidative stress and maintaining intracellular redox balance 9. Prior evidence revealed that glucose-6-phosphate dehydrogenase (G6PD) provides neuroprotective effects by enhancing the oxidative pentose phosphate pathway (PPP) 7, 9-11. One-carbon (1C) metabolism refers to a complex biochemical network involving pathways such as the folate and methionine cycles, which also serve as critical suppliers of essential components (i.e., purines, pyrimidines, S-adenosylmethionine, and NADPH) to support cell growth and proliferation 12-14. Traditionally, 1C metabolism is believed to primarily provide 1C units rather than NADPH 12-14. However, silencing critical genes involved in 1C metabolism, such as SHMT2 and MTHFD2, reduce the NADPH/NADP^+^ and reduced/oxidized glutathione ratios (GSH/GSSG) in cells, compromising the oxidative stress response 15. However, the mechanisms underlying the disturbance of 1C metabolism after ICH and its implications in neuronal damage remain poorly understood.

The endoplasmic reticulum (ER) maintains protein equilibrium but is susceptible to stress. Prolonged endoplasmic reticulum stress (ERS) can trigger adaptive responses called the unfolded protein response (UPR), which primarily involves the PERK/ATF4, IRE1α/XBP1s, and ATF6 signaling pathways 16. GRP78 is an upstream effector of the three ERS pathways and plays a central role in regulating the ERS^17^, ^18^. Recent research indicates that the ER acts as a quality-control organelle for proteins and is implicated in processes such as autophagy, oxidative stress, and metabolism. Notably, the PERK pathway has been shown to be essential in modulating various aspects of protein synthesis, including protein functions and mass 19. Furthermore, studies have reported that the absence or mutations of PERK at its phosphorylation site in eIF2α caused impaired glucose metabolism in mice 20-22. Additionally, it has been demonstrated that ATF4 not only regulates SHMT2 and other genes associated with 1C metabolism but also sustains mitochondrial homeostasis and protects cells from ROS-induced cell death 23-25. However, it remains unclear whether there is an interaction between the PERK/ATF4 pathway and mitochondrial 1C metabolism in maintaining cellular metabolism and redox balance following ICH.

Hemin is a degradation product of hemoglobin released during hemorrhagic events within the brain and contributes to the activation of secondary injury mechanisms, such as oxidative stress, inflammation, and neuronal damage 26. Recent studies have reported that exposure of cultured neurons to hemin can be used to reliably model the effects of hemorrhagic stroke in rodent models 27, 28. Therefore, in this study, we used a hemin-induced primary neuronal injury model to investigate the mechanisms underlying neuronal injury after ICH.

Herein, our investigation, for the first time, revealed the upregulation of PERK/ATF4 and 1C metabolism signals in hemin-treated neurons through metabolomic and transcriptomic analyses. Our findings showed that 1C metabolism genes are necessary to maintain mitochondrial function, morphology, and cellular redox homeostasis, which are also directly regulated by the PERK/ATF4 branch of the UPR. In addition, supplementation with exogenous NADPH after ICH effectively rescued neuronal apoptosis, and an interaction was revealed between MTHFD2, a key player in mitochondrial 1C metabolism, and GRP78, which in turn reversely regulates the PERK/ATF4 branch to ensure ER homeostasis.

## Materials and Methods

### *In vivo* model of ICH

C57BL/6 mice (20-25 g, 8-12 weeks; GemPharmatech, Jiang Su, China) were used in this study, and all animal experiments followed the recommended guidelines and were approved by the Institutional Animal Care and Use Committee of the School of Biomedical Engineering, Shanghai Jiao Tong University. The mice were kept in sterile conditions at 20-25 °C and 40-50% humidity with 12 h light-dark cycles. For the ICH procedure, mice were immobilized using a stereotactic framework (RWD, Shenzhen, China), administered collagenase IV (Sigma, USA) 29, and then randomly allocated into control, ICH, ICH+GSK2606414, and ICH+NADPH groups (n=8 per group). In the ICH+GSK2606414 group, the PERK inhibitor, GSK2606414 (50 mg/kg/day), was orally administered daily for 6 days post-ICH. In the ICH+NADPH group, an intraperitoneal injection of NADPH (5 mg/kg/day) was administered daily until 6 days post-ICH. After deep anesthesia, the mice were perfused with phosphate-buffered saline (PBS) at 12 h, 1 d, 3 d, and 7 d post-ICH and then fixed with 4% paraformaldehyde (PFA). Next, the brains were dehydrated in 30% sucrose, and 20 μm brain sections were prepared and subjected to immunofluorescence (IF) staining. They were euthanized at 12 h, 1 d, 3 d, and 7 d postoperatively, and the tissues surrounding the lesion site were collected to prepare lysates for subsequent western blot experiments.

### Isolation and culturing of primary neurons from mouse cortex and hippocampus

Primary neurons were retrieved from the cortex and hippocampus of C57BL/6 embryonic mice on embryonic days 16-18, digested with 0.125% trypsin for 5-10 min, followed by gentle dispersion. The resulting suspensions were passed through a 40-μm filter and grown in a neurobasal medium (21103049, Gibco, USA) with 2% B27 (17504044, Gibco, USA), 1% GlutaMAX (35050061, Gibco, USA), and 0.5% penicillin/streptomycin. We quantified neurons, seeded them on poly-D-lysine-coated culture plates, and cultured them at 37 °C with 5% CO_2_. Half of the culture medium was replenished after 2-3 days, and the neurons were used for experiments after 5-7 days of culture.

### Cell viability assay

To overcome the challenges in maintaining neuronal viability in 96-well plates, neurons were cultured in 24- or 12-well plates for 5-7 days. Briefly, the cells were incubated with a CCK-8 solution (10% v/v in culture medium) for 2 h at 37 °C in a 5% CO_2_ atmosphere. To evaluate cell viability, absorbance was measured at 450 nm using a BioTek microplate reader. Each experiment was performed in triplicate to ensure statistical robustness.

### Immunofluorescence assay

Neurons cultured on coverslips were fixed with 4% PFA and permeated with 0.3% Triton X-100 for 10 min. After blocking with 10% bovine serum albumin at room temperature for at least 1 h, they were incubated overnight at 4 °C with primary antibodies, including GRP78 (66574-1-lg, 1:200), SHMT2 (11099-1-AP, 1:200), ALDH1L2 (21391-1-AP, 1:200), and VDAC1 (66345-1-Ig, 1:200) purchased from Proteintech, as well as MTHFD2 (ab307428, 1:200) from Abcam and NeuN (AG5317, 1:50) from Beyotime. Subsequently, the cells were treated with Alexa Fluor-conjugated secondary antibodies at 37 °C for 1-2 h and stained with DAPI to visualize the nuclei. Donkey anti-rabbit Alexa Fluor 555 (A0453; 1:200) and goat anti-mouse Alexa Fluor 488 (A0428; 1:200) antibodies were purchased from Beyotime. Finally, slides were mounted using an anti-fade mounting medium and examined under a fluorescence microscope.

### TUNEL staining

A TUNEL assay (Beyotime, China) was performed to assess neuronal apoptosis. Primary neurons and brain sections were fixed in 4% PFA for 10 min and infiltrated with 0.3% Triton X-100 for 10 min. After washing thoroughly with PBS, the cells were stained with DAPI to visualize the nuclei, and mounted according to the manufacturer's instructions. Fluorescence was observed under a fluorescence microscope, and images were captured for analysis.

### Western blotting and co-immunoprecipitation assay

Total protein from primary neurons or mouse brain tissue was retrieved and analyzed using a BCA Protein Assay Kit (Meilunbio, China). The proteins were electrophoresed and transferred onto polyvinylidene fluoride membranes, and incubated at 4 °C overnight with primary antibodies against GRP78 (66574-1-lg, 1:5000), PERK (24390-1-AP; 1:1000), eIF2α (11170-1-AP; 1:5000), p-eIF2α (28740-1-AP, 1:1500), CHOP (66741-1-Ig; 1:2000), PSPH (14513-1-AP, 1:1500), PSAT1 (10501-1-AP, 1:5000), BAX (50599-2-Ig, 1:3000), PHGDH (14719-1-AP, 1:3000), Fis1 (10956-1-AP, 1:1500), caspase3 (66470-2-Ig, 1:1500) and Bcl2 (26593-1-AP, 1:5000), which were purchased from Proteintech, as well as antibodies against puromycin (Kf-Ab02366-1.1, 1:1000; Kerafast), MTHFD2 (ab307428, 1:1000, Abcam), DRP1 (K009368P, 1:1000; Solarbio), phospho-DRP1(Ser616) (abs137991, 1:1000; Absin), SHMT2 (33443S, 1:1000; Cell Signaling), XBP1s (40435S, 1:1000; Cell Signaling), MFN1 (AF7461, 1:2000; Beyotime), β-actin (AF0003, 1:1000; Beyotime), MFN2 (AF7473, 1:2000; Beyotime), ATF4 (AF2560, 1:1000; Beyotime), ATF6 (AF6243, 1:1500; Beyotime), and p-PERK (AP328, 1:1000; Beyotime). Then, for the detection process, the membranes were treated with horseradish peroxidase-conjugated secondary antibodies at room temperature before using the Super Sensitive Chemiluminescence Kit (Meilunbio, China). Bands were identified using an eBlot Touch Imager with ECL reagent, following the manufacturer's instructions. β-actin served as the control. In the co-immunoprecipitation (Co-IP) experiment, lysates from around 10 million primary neurons were immunoprecipitated with protein A/G magnetic beads linked to the appropriate antibody within the IP buffer. Protein interactions were subsequently analyzed through western blot.

### Electron microscopy

Primary neurons retrieved from the cortex and hippocampus of C57BL/6 mice at embryonic days 16-18, were centrifuged at 800 rpm for 5 min and immediately fixed in 2.5% glutaraldehyde at 4 °C for 6 h. They were then fixed with 1% osmium tetroxide, dehydrated using different ethanol concentration gradients, and embedded in araldite. Thin sections (60-nm) were stained with 2.0% uranyl acetate and lead citrate before an FEI electron microscope analysis. Each experimental group comprised three replicates.

### RNA extraction and qRT-PCR

Primary neuronal RNA extraction was performed using TRIzol reagent. NanoDrop ND-1000 was used to determine the total RNA quantity. Briefly, 1 μg of total RNA was reverse-transcribed into cDNA using a PrimeScript RT reagent kit. qRT-PCR was conducted using the SYBR Green Master Mix in a thermal cycler with specific primers (**Supplementary Table S1**). The cycling conditions entailed an initial denaturation step at 95 °C for 5 min, followed by 40 cycles of denaturation at 95 °C for 15 s and annealing at 60 °C for 1 min. Subsequently, melting curve analysis was conducted to verify the specificity of PCR amplification. GAPDH served as the internal control.

### RNA sequencing

Primary neurons treated with hemin (sc-202646, Santa Cruz Biotechnology, USA) and those with SHMT2, MTHFD2, and ALDH1L2 knockdown were collected, and RNA was extracted using TRIzol. RNA quality was evaluated using a Bioanalyzer 2100, and RNA samples of high quality, with an RNA integrity number > 7, were chosen for library preparation. The libraries were then sequenced on an Illumina NovaSeq 6000 system (Biotree Biotech, China), resulting in 150 bp paired-end reads. Subsequently, high-quality reads were obtained by filtering using the CutAdapt software. The corresponding RNA sequencing data were deposited in the GEO database under accession numbers GSE263227 and GSE263244.

### CUT&Tag sequencing and Chromatin immunoprecipitation (ChIP)-qPCR assay

CUT&Tag sequencing was performed according to established protocols 30, starting with approximately 100,000 neurons washed twice with the wash buffer. Then, 10 μL of concanavalin A-coated magnetic beads were added and incubated for 10 min at room temperature. After discarding the unbound supernatant, the bead-bound neurons were resuspended in dig wash buffer with either ATF4 primary antibody (11815S, 1:50, Cell Signaling Technology) or IgG control antibody and incubated overnight at 4 °C on a rotating platform. The primary antibody was then removed, and the neurons were incubated with a secondary antibody (AP132, 1:100, Millipore) at room temperature for 60 min. This was followed by incubation with the pA-Tn5 adapter complex in a Dig-med buffer at room temperature for approximately 1 h. DNA was extracted and sequenced using an Illumina NovaSeq 6000 following the manufacturer's guidelines. The corresponding CUT&Tag sequencing data were submitted to the GEO Omnibus (GSE263464) database.

For ChIP-qPCR, we used a ChIP Assay Kit (Beyotime, China). At least 1×10^7^ HT22 cells were fixed in 1% formaldehyde for 10 min at 37 °C. The fixed cells were harvested, lysed, and sonicated using Sonics Vibra Cell (Sonics, USA). Immunoprecipitation was performed at 4 °C overnight with anti-ATF4 (11815S, 1:200, Cell Signaling Technology) or a normal rabbit IgG antibody. The precipitated DNA was amplified by PCR. **Supplementary Table S2** lists the primer sequences used for the ChIP assay.

### Flow cytometry analysis

Annexin V-APC/7-AAD double staining was used to detect apoptosis in the primary neurons. Neurons were washed twice with calcium-free Dulbecco's PBS and resuspended in a binding buffer. They were then incubated with 5 µl of Annexin V-APC and 5 µl of 7-AAD in the dark at room temperature for approximately 20 min. Flow cytometry (BECKMAN COULTER, USA) was used to determine the percentage of neurons in early apoptosis (Annexin V-APC positive/7-AAD negative) and late apoptosis (Annexin V-APC positive/7-AAD positive). Additionally, the mitochondrial membrane potential (MMP) was assessed using TMRE (Beyotime, China); the neurons were incubated in 1× TMRE for 30 min in the dark at room temperature, using CCCP as a positive control to induce a reduction in MMP.

### Measurement of NADPH/NADP^+^ ratio and ATP content

Briefly, cells were lysed according to the manufacturer's protocol, and the resulting lysate was used to quantify NADP⁺ and NADPH levels. The optical density was assayed at 450 nm, and the NADPH/NADP⁺ ratio was determined using a NADP⁺/NADPH assay kit (Beyotime, China) by referencing a standard curve. The ATP content was precisely quantified using an ATP Assay Kit (Beyotime, China).

### Mitochondrial superoxide, membrane potential and feature measurements

Superoxide levels in neuronal mitochondria were determined using the MitoSOX Red indicator (Yeasen, China). Briefly, MMP was assessed using an MMP kit and TMRE (Beyotime, China). Laser scanning confocal microscopy was used to obtain the corresponding fluorescence images.

To assess mitochondrial morphology and fluorescence intensity, confocal laser scanning microscopy with a 60× oil immersion objective (LEICA SP5, Germany) was employed to capture the images. The mean mitochondrial area, branch length, and fluorescence intensity were analyzed using ImageJ software. The "Mitochondria Analyzer" was employed to quantify mitochondrial morphology, allowing for the detailed examination of mitochondrial structure, including mitochondrial length and branch length.

### Non-targeted metabolite extraction and analysis

To investigate the potential metabolic alterations induced by hemin treatment in primary neurons, we collected six samples from the control and hemin-treated groups, with each sample containing at least 5×10^6^ primary neurons, which were rapidly frozen in liquid nitrogen for subsequent analysis. Non-targeted metabolites were determined using the Shanghai Biotree Biotech Platform. Detailed procedures for sample extraction and measurements are provided in the **Supplementary Method S1**. LC-MS/MS was performed using an ultra-high-performance liquid chromatography system (Vanquish, Thermo Fisher Scientific, USA). Raw data were used for principal component analysis (PCA) using SIMCA V16.0.2. Statistical significance was determined based on a fold-change (FC) > 2 and *p* < 0.05.

### Targeted metabolite extraction and analysis

Sample processing included a two-step metabolite analysis approach, in which non-targeted extraction of metabolites was performed, followed by targeted analysis of metabolites on the Shanghai Metabo-Profile Biotech platform. We focused on detecting metabolites involved in one-carbon (1C) metabolism using ultra-performance liquid chromatography coupled with tandem mass spectrometry (UPLC-MS/MS). Detailed metabolite extraction and analysis protocols are provided in the **Supplementary Method S2**. The raw UPLC-MS/MS data were analyzed using the MassLynx software, and statistical significance was set at FC ≥ 2 and a *p* ≤ 0.05.

### Histological analyses

Mouse brain tissues were perfused with PBS and fixed overnight in 4% PFA. Subsequently, 20 μm-thick brain sections were prepared through sucrose gradients. For staining, the sections were incubated in hematoxylin for 5 min, rinsed in water for 10 min, incubated in 0.5% eosin (HE) solution for 2 min, briefly rinsed in water, and dehydrated twice in 70% ethanol. Following a procedure similar to that used for hematoxylin and HE staining, the sections were stained with 0.1% cresyl violet for 3 min.

### Lentiviral vector transfection

To downregulate or upregulate the expression levels of specific target genes in primary neurons, a lentiviral packaging system was employed to achieve stable knockdown or overexpression of these genes (OBiO Biotech, Shanghai, China). The sequences for the specific shRNAs used for gene silencing are detailed in **Supplementary Table S3**. A negative control shRNA (NC shRNA) was also engineered. In parallel, ATF4 and MTHFD2 were overexpressed using lentiviral vectors designated as pcSLenti-EF1-EGFP-CMV-Atf4-3xFLAG-WPREC and pcSLenti-EF1-EGFP-CMV-Mthfd2-3xFLAG-WPREC, respectively.

### Assessment of transfection efficiency

The efficiency of the lentiviral transfection was evaluated using multiple approaches to ensure accurate and reliable quantification. First, the lentiviral vectors used for transfection included an enhanced green fluorescent protein (EGFP) reporter gene, allowing visualization of transfection efficiency under a fluorescence microscope. Transfection efficiency was determined by calculating the percentage of EGFP-positive cells relative to the total cell population, with a multiplicity of infection (MOI) of 10, achieving an efficiency of approximately 90% (**Supplementary Figure S5A**).

In addition to fluorescence microscopy, qRT-PCR and western blotting were performed to assess the knockdown efficiency of target genes. qRT-PCR was used to quantify the mRNA levels of the target genes, confirming a significant reduction to 5-30% of the control levels post-transfection. Western blot analysis was performed to evaluate protein expression, further validating the knockdown efficiency at the mRNA level.

### Neurological scoring

Neurological function was evaluated in each mouse at 1, 3, 7, and 14 d post-ICH using a suite of neurobehavioral tests: modified neurological severity score (mNSS), rotarod test, and elevated body swing test (EBST). The mNSS is a comprehensive assessment tool to quantify neurological impairments on a scale from 0 (normal) to 14 (maximum deficit). It includes tests for limb flexion during tail elevation (0-3 points), walking posture (0-3 points), balance on a beam (0-6 points), and pinna and corneal reflexes (0-2 points), where higher scores correspond to more severe neurological damage 31, 32.

Motor coordination and balance were tested using the rotarod test, which began with a 3-day pre-ICH training period. Initially, mice were placed on a stationary rod for 1 min for acclimatization. The speed of the rod was gradually increased to 20 rotations per minute (rpm) and maintained for 5 min. On the final training day, the mice underwent two baseline tests and the rod was accelerated to 40 rpm, and their ability to remain on the rod for up to 5 min was noted along with their fall rate 32.

The EBST measures unilateral motor function by lifting mice with their tails to observe the direction of body swings. Over 20 trials, each swing's direction (right or left) was recorded and the percentage of swings biased to one side was calculated to assess any asymmetry in motor function 33.

### ICH volume measurement

Mice (n=6/group) were euthanized using 7% chloral hydrate three days after ICH. Their brains were rapidly excised and preserved in isopentane by rapid freezing. Subsequently, brain sections (1 mm thick) were prepared using a stainless-steel mouse brain matrix and photographed. ImageJ software was used to determine the hematoma size. The hematoma volume was calculated by summing the hemorrhage area from each section and multiplying it by the section thickness (1 mm).

### Statistical analysis

GraphPad Prism 8 software was used for the statistical analysis of parametric data. To compare the differences, mean mitochondrial branch length, and mean area between the two groups (continuous variables), Student's *t*-test was applied. For comparisons involving more than two groups, one-way analysis of variance (ANOVA) was used to evaluate the statistical significance between each group, followed by post-hoc comparisons using Tukey's multiple comparison test because of its ability to control for Type I errors across multiple comparisons. For experiments involving data across more than one independent variable, two-way ANOVA was employed to evaluate the effects of different treatments over time or across different conditions. Statistical significance was set at *p* < 0.05.

## Results

### *In vitro* and *in vivo* ICH models

**Figure 1:**
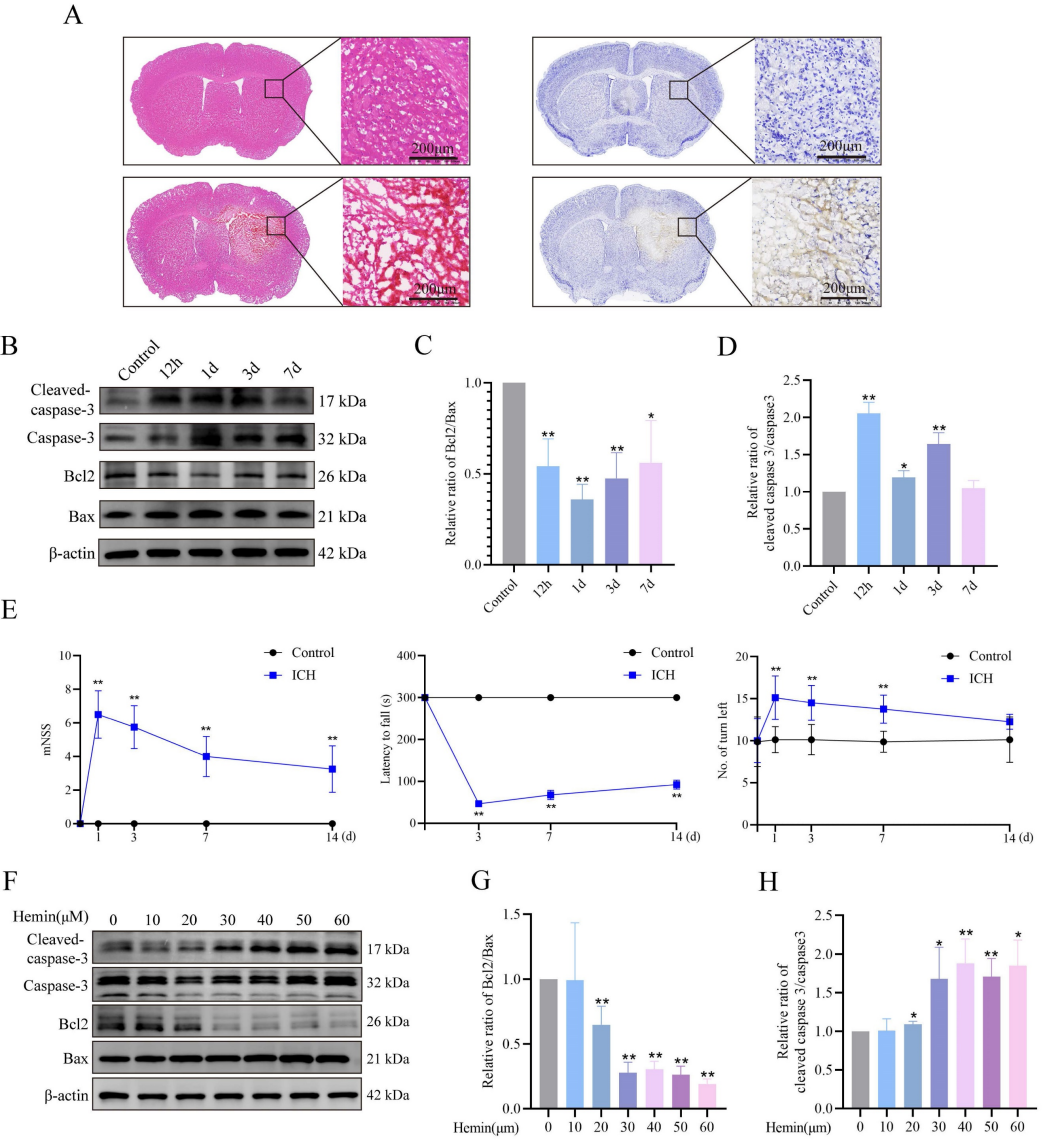
** ICH-induced neuronal apoptosis and neurological dysfunction. A)** Representative photomicrographs of HE- and Nissl-stained brain tissues in the ICH group.** B)** Western blot analysis of apoptosis markers in the protein extracts from mouse perihematomal brain tissues at different times after ICH. **C-D)** Bcl2/Bax expression levels and cleaved-caspase3/caspase3 protein ratio in each group (n = 3/group). **E)** mNSS, rotarod test, and EBST results to evaluate the neurobehavioral functions of mice at different times (n = 8/group). **F)** Expression of apoptosis markers in the primary neurons treated with different concentrations of hemin for 24 h. **G-H)** Bcl2/Bax expression levels and cleaved-caspase3/caspase3 protein ratios in each group (n = 3/group). Data are presented as mean ± SD. * *p* < 0.05, ** *p* < 0.01.

We established ICH mouse models and confirmed the occurrence of neuronal apoptosis following ICH induction. We conducted HE and Nissl staining (cresyl violet staining) to assess neuronal morphology and observed shrunken, dark neuronal nuclei, and neuronal disarrangement in the ICH group (**Figure 1A**). Western blot analysis revealed a notable decrease in the Bcl2/Bax protein ratio and a simultaneous increase in the ICH mouse group's cleaved-caspase3/caspase3 protein ratio (**Figure 1B-D**). Furthermore, we evaluated the neurological function of mice post-ICH using behavioral tests, including the mNSS, rotarod test, and EBST. Both the mNSS and EBST demonstrated significant neurological deficits after ICH. The rotarod test, which was used to assess motor function, showed that ICH group mice spent less time on the rotarod compared to the control group (**Figure 1E**). To simulate *in vivo* conditions, we established a model of hemin-induced primary neuronal damage. The purity of the primary cultured neurons, assessed by MAP2 expression, exceeded 90% (**Supplementary Figure S1A**). Cell viability was assessed following a 24-h treatment period using different hemin concentrations. The results demonstrated a dose-dependent effect of hemin on cell viability (**Supplementary Figure S1B**). Notably, at a concentration of 30 μM, hemin led to a significant 30% reduction in cell viability, accompanied by alterations in the Bcl2/Bax and cleaved-caspase3/caspase3 protein ratios compared to the control group (**Figure 1F-H**). However, concentrations exceeding 30 μM resulted in severe neuronal damage and synaptic cleavage, which may not accurately reflect the stress changes during injury (**Supplementary Figure S1C**). Hence, in subsequent experiments, we selected 30 μM as the optimal stimulation concentration for primary neurons *in vitro*. These findings confirmed the successful establishment of ICH damage in animal and cellular models.

### Integration of transcriptomic and metabolomics analyses of primary neurons treated with hemin

To investigate the impact of hemin on gene expression in neurons, we examined changes in the transcriptomics and metabolomics of primary neurons following 24 h of hemin treatment. RNA sequencing and non-targeted metabolomic analyses were performed.

**Figure 2:**
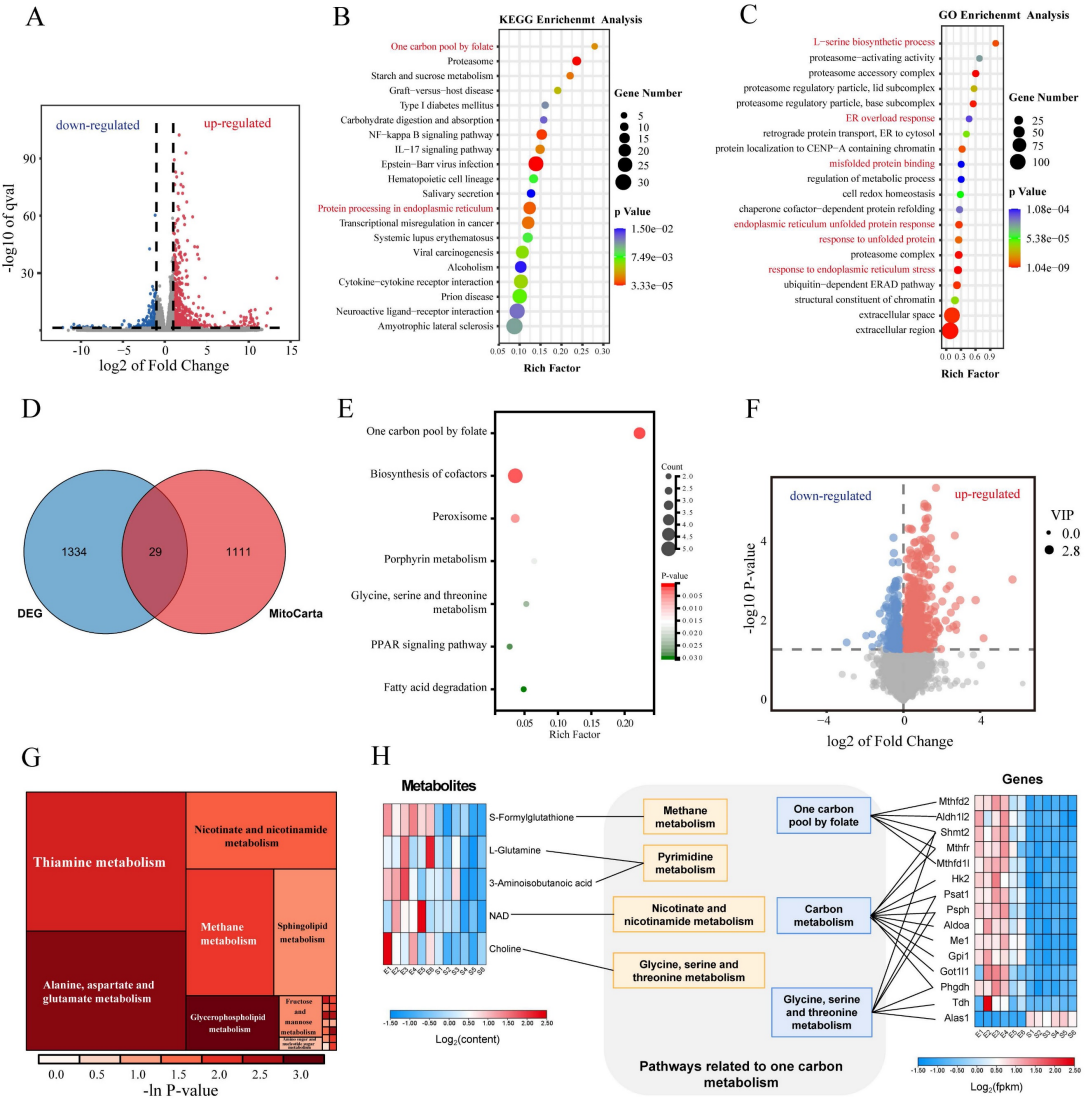
** Integration of RNA sequencing and metabolomics in primary neurons treated with hemin for 24 h. A)** Volcano plot illustrating differences in gene expression between control neurons and those treated with hemin for 24 h (*p* < 0.05 and log_2_FC≥1).** B-C)** Gene set enrichment analysis of KEGG and GO gene sets based on the fold change of gene expression in the hemin-treated group. Significantly enriched gene sets (*p* < 0.05) were identified, and the selected gene sets are highlighted in red font. **D)** Venn diagrams showing the number of differentially expressed genes that overlap with MitoCarta3.0. **E)** KEGG analysis of the 29 differentially expressed genes that overlap with MitoCarta3.0. **F)** Volcano plot depicting differentially expressed metabolites between the control group and the hemin-treated group (*p* < 0.05, VIP value > 1). **G)** Rectangular tree diagram for enrichment analysis of differential metabolite metabolic pathways. The size of the square indicates the pathway's impact on the topology analysis, and the color represents the p-value of the enrichment analysis. **H)** Joint pathway analysis of significantly altered genes (RNA-sequencing, right) and metabolites (metabolomics, left).

Transcriptomic alterations after hemin treatment were assessed, with differential gene screening criteria set at FC ≥ 2 and p ≤ 0.05, revealing 1,363 DEGs, among which 1,071 and 292 genes were upregulated and downregulated, respectively (**Figure 2A**). GO and KEGG enrichment analyses revealed dysfunction in ER function in the hemin-treated group (**Figure 2B, C**). Notably, pathways associated with 1C metabolism, including "one carbon pool by folate" and "L-serine biosynthetic processes," ranked among the top 20 upregulated gene sets in both KEGG and GO enrichment analyses (**Figure 2B, C**). At the transcriptional level, we investigated the specific mitochondrial processes that were affected in hemin-treated neurons. We analyzed all differentially expressed mitochondria-related genes using the MitoCarta3.0 database, which encompasses all mitochondria-related genes, and conducted KEGG enrichment analysis (**Figure 2D, Supplementary Figure S2A**) 34. KEGG analysis of mitochondria-related differential genes revealed that the 1C metabolism pathway was the most significantly disrupted (**Figure 2E**). Non-targeted metabolomic analysis was performed to observe global metabolic profile changes following hemin treatment in primary neurons.

PCA, supervised orthogonal partial least squares discriminant analysis (OPLS-DA), and permutation plot tests of the OPLS-DA models revealed notable differences in the distribution of metabolites between the control and hemin-treated groups (**Supplementary Figure S2B-H**). Differences in metabolite proportions were observed according to a VIP value >1 in the OPLS-DA model and *p*<0.05, as determined by Student's *t*-test between the control and hemin-treated groups (**Figure 2F**). KEGG enrichment analysis (**Figure 2G**) highlighted the enriched pathways. To comprehensively analyze the metabolic profile changes induced by hemin, we conducted integrated transcriptomic and metabolomic analyses to compare the control group with those treated with hemin (**Figure 2H**). Taken together, our transcriptomic and metabolomic analyses indicate that hemin induces ERS and disrupts 1C metabolism in neurons.

### Hemin-induced reprogramming of 1C metabolism and ERS in primary neurons

**Figure 3:**
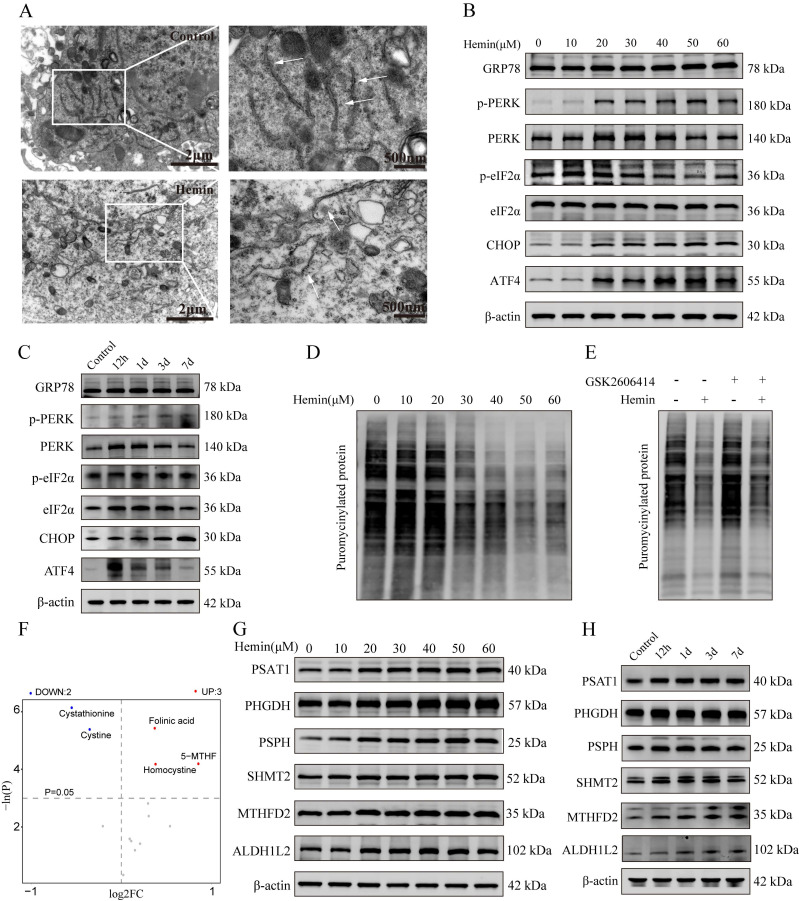
** Hemin-induced ERS and one-carbon metabolic remodeling in primary neurons. A)** Representative TEM images depicting ERS in primary neurons treated with hemin. **B)** Western blot analysis of ERS markers in neurons following treatment with various concentrations of hemin for 24 h. **C)** Western blot analysis of ERS markers in protein extracted from perihematomal brain tissues of mice at different time points after ICH. **D)** Primary neurons were cultured, treated with various concentrations of hemin for 24 h, and subsequently exposed to 1 μM puromycin for 0.5 h before harvesting. **E)** Primary neurons were exposed to 30 μM hemin and/or 0.4 nM GSK2606414 for 24 h, followed by treatment with 1 μM puromycin for 0.5 h before harvest to assess protein synthesis using western blot analysis. **F)** Volcano plot displaying differentially expressed metabolites associated with one-carbon metabolism between the control and hemin-treated groups identified by targeted metabolomics (*p* < 0.05). **G)** Western blot was used to detect the expression of proteins related to one-carbon metabolism in primary neurons treated with various concentrations of hemin for 24 h. **H)** Protein extracted from perihematomal brain tissues of mice at different time points after ICH was subjected to western blot analysis to assess the expression of proteins related to one-carbon metabolism.

To validate the induction of ERS in hemin-treated neurons, their ultrastructure was assessed using a transmission electron microscope (**Figure 3A**), which revealed abnormal expansion and swelling of the ER and loss of the normal folded structure, indicating disrupted ER homeostasis in hemin-treated neurons. Western blotting results showed upregulated expression of markers associated with ERS in both hemin-treated neurons and adjacent hematomas in mouse brain tissues following ICH (**Figure 3B, C**). Statistical analyses of the western blot results are shown in the bar chart in **Supplementary Figure S3A-D**. Furthermore, the mRNA expression levels were validated using qRT-PCR (**Supplementary Figure S3E**). Additionally, hemin inhibited puromycin-labeled protein synthesis in a dose-dependent manner, which could be reversed by the PERK inhibitor GSK2606414 (**Figure 3D, E**). These results indicate that hemin can activate the PERK/ATF4 branch of UPR.

To explore the changes in metabolites associated with 1C metabolism, we conducted a targeted metabolomic analysis to compare the 1C metabolism profiles between the control and hemin-treated groups. A comprehensive list of all identified metabolites related to 1C metabolism is provided in **Supplementary Table S4**. Similar to the non-targeted metabolomic analysis, PCA, OPLS-DA, and permutation tests of the OPLS-DA model demonstrated significant differences in the metabolite distribution between the hemin-treated and control groups (**Supplementary Figure S3F-J**). Based on an FC greater than 2 and a p-value less than 0.05, we identified three compounds that increased and two compounds that decreased in the hemin-treated group compared to the control (**Figure 3F**). Furthermore, we assessed the expression of proteins associated with 1C metabolism in mouse brain tissues following ICH and in hemin-treated primary neurons and found elevated levels of proteins related to 1C metabolism in hemin-treated primary neurons and brain tissue adjacent to the hematoma (**Figure 3G, H**). The corresponding statistical assessments of the western blots are shown in **Supplementary Figure S4A, B**. Collectively, these results confirm the occurrence of ERS and metabolic reprogramming of 1C metabolism after ICH.

### PERK/ATF4 pathway transcriptionally activates 1C metabolism genes via ATF4

**Figure 4:**
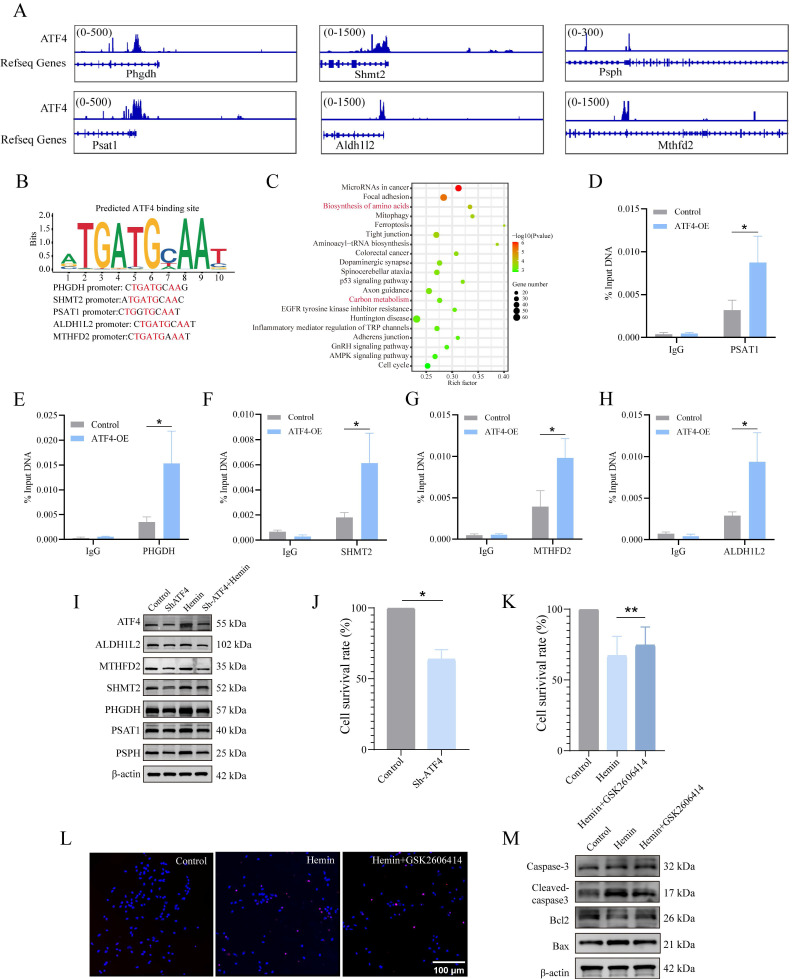
** PERK/ATF4 branch of ERS transcriptionally activates 1C metabolism genes through ATF4. A)** Genome Browser views depicting CUT&Tag signal ATF4-binding peaks in regions proximal to transcription start sites of* Shmt2, Mthfd2, Aldh1l2, Phgdh, Psat1* and* Psph*. **B)** ATF4 binding site in the promoter region of 1C metabolism genes. **C)** GO analysis of ATF4 binding peaks at target genes. Gene sets related to 1C metabolism are highlighted in red font. **D-H)** ChIP-qPCR was used to detect the binding of ATF4 to the promoter region of *Psat1, Phgdh, Shmt2, Mthfd2 and Aldh1l2* in HT22 cells, and IgG was applied as a negative control. **I)** Expression of proteins related to 1C metabolism in primary neurons after ATF4 knockdown. **J)** Neuronal viability evaluated using the CCK-8 kit after ATF4 knockdown (n = 3/group). **K)** Neuronal viability assessed using the CCK-8 kit in cells treated with or without GSK2606414 for 24 h (n = 3/group). **L)** Representative confocal images of TUNEL staining in primary neurons treated with or without GSK2606414 for 24 h. **M)** Western blot analysis of the apoptosis marker in primary neurons treated with or without GSK2606414 for 24 h. Data are presented as mean ± SD. * *p* < 0.05, ** *p* < 0.01.

As mentioned previously, both 1C metabolism and the ERS undergo significant alterations following ICH. However, whether these two cellular processes exert coordinated effects on each other remains unclear. Given that ATF4 is a crucial transcription factor in antioxidant reactions and amino acid metabolism, we investigated whether the PERK/ATF4 branch of the ERS plays a regulatory role in 1C metabolism. Therefore, we used CUT&Tag analysis to identify potential genes regulated by ATF4 in neurons. The CUT&Tag results showed that the peaks of 1C metabolism genes, including *Phgdh, Psat1, Psph, Shmt2, Mthfd2, and Aldh1l2*, were significantly enriched in the neurons (**Figure 4A**). We identified a putative ATF4 binding site in the promoter region of 1C metabolism genes (**Figure 4B**). The enrichment bubble map of peaks (representing significantly enriched reads) associated with adjacent gene pathways highlighted the top 20 upregulated gene sets, with those related to 1C metabolism shown in red (**Figure 4C**). ChIP-seq databases (GSE35681, GSE75165, and GSE44338) were also investigated, revealing firm binding peaks of ATF4 in the genomic regions of 1C metabolism genes (**Supplementary Figure S4C-E**). Moreover, we generated overexpressed (OE) ATF4 HT22 cell lines (ATF4-OE), and ChIP-qPCR further confirmed that ATF4 could bind to the promoter regions of *Psat1, Phgdh, Shmt2, Mthfd2 and Aldh1l2* (**Figure 4D-H**) in HT22 cells. These data demonstrate that ATF4 activates *Psat1, Phgdh, Shmt2, Mthfd2, and Aldh1l2* by binding to their promoters. Next, we investigated the function of ATF4 in regulating 1C metabolism genes by downregulating ATF4 expression in neurons using a lentivirus. At an MOI of 10, the transfection efficiency of the lentivirus approached 90% (**Supplementary Figure S5A**). Taken together, these results demonstrated that upon neuronal ATF4 downregulation, there was a significant decrease in both the transcription and protein levels of 1C metabolic genes (*Psat1, Phgdh, Psph, Shmt2, Mthfd2 and Aldh1l2*) (**Figure 4I, Supplementary Figure S5B, C**), which suggests that ATF4 modulates the expression of 1C metabolism genes.

ATF4 is a versatile transcription factor that is pivotal in regulating various cellular processes and maintaining cell function. However, during prolonged ERS, ATF4 can also stimulate CHOP transcription 35, 36, which initiates an apoptotic cascade. Therefore, we assessed the role of ATF4 in cell survival and observed that the knockdown of ATF4 reduced cell viability compared to that in the control group under normal conditions **(Figure 4J)**. Furthermore, we explored the impact of a drug inhibitor of the PERK/ATF4 axis, GSK2606414, on the neurotoxicity of hemin-treated neurons (**Supplementary Figure S5D**). GSK2606414 was administered to primary neurons 30 min before 24 h of hemin stimulation, and we evaluated cell viability, conducted TUNEL assays, and assessed the expression of apoptosis-related proteins. The results of the CCK8 assay indicated that GSK2606414 treatment significantly improved cell viability compared to the hemin-treated group **(Figure 4K)**. TUNEL assay results suggested that GSK2606414 inhibited apoptosis in hemin-treated neurons (**Figure 4L, Supplementary Figure S5E**). Additionally, western blotting experiments confirmed the inhibitory effect of GSK2606414 on apoptosis in hemin-treated neurons based on alterations in the corresponding proteins (**Figure 4M, Supplementary Figure S5F**). Collectively, these findings suggest that under normal physiological conditions, the PERK/ATF4 axis initiates pro-survival signaling. However, under severe stress conditions, such as ICH, the PERK/ATF4 axis may switch to initiate pro-apoptotic signaling.

### Mitochondrial 1C metabolism is necessary for maintaining mitochondrial function, morphology, and redox homeostasis

**Figure 5:**
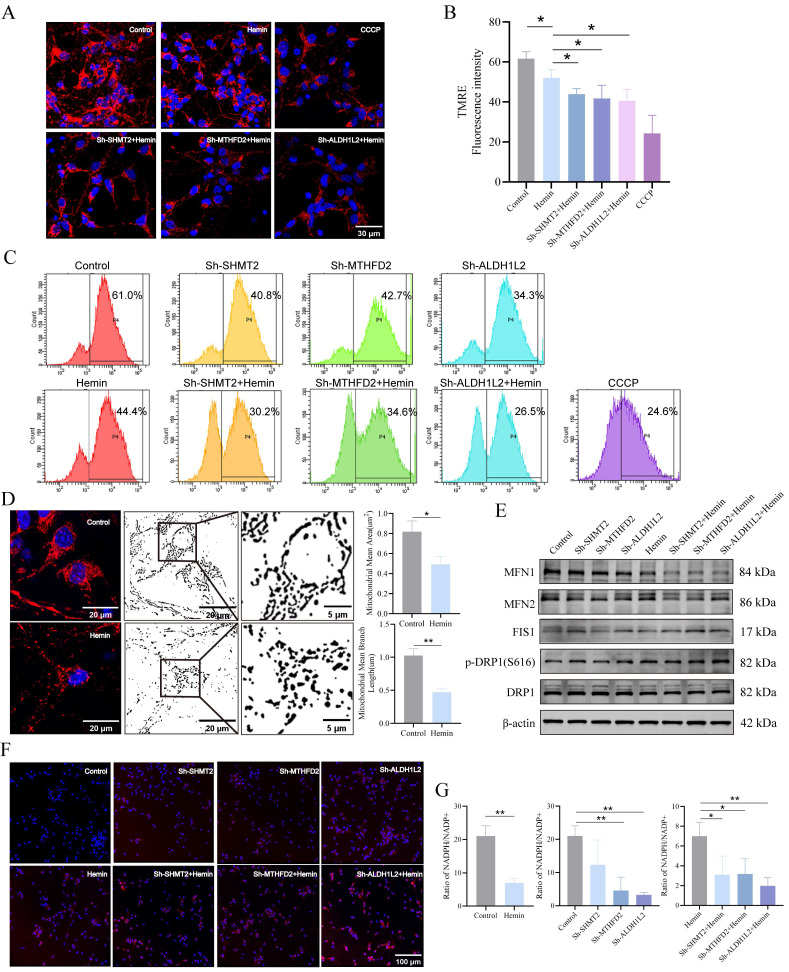
** SHMT2, MTHFD2, and ALDH1L2 are necessary for maintaining mitochondrial function, morphology, and redox homeostasis. A)** Mitochondrial membrane potential (MMP) in SHMT2, MTHFD2, and ALDH1L2 knocked-down primary neurons treated with hemin for 24 h using TMRE staining. **B)** Quantification of red fluorescence intensity indicating changes in SHMT2, MTHFD2, and ALDH1L2 knocked-down primary neurons after treatment with hemin for 24 h (n = 3/group). **C)** MMPs assessed via flow cytometry and TMRE staining, with CCCP as the positive control. **D)** Confocal microscopy images displaying mitochondrial fragmentation in the hemin-treated group using MitoTracker staining (n = 3/group). Quantitative image analysis of mitochondrial mean branch length (um) and mitochondrial mean area (um^2^) was performed by ImageJ software. **E)** Representative western blot analysis of MFN1, MFN2, FIS1, DRP1, and p-DRP1 (S616) in the control group, hemin-treated group, and SHMT2, MTHFD2 or ALDH1L2-knocked down primary neurons. **F)** Mitochondrial superoxide levels in primary neurons using MitoSOX staining. **G)** Evaluation of the cellular NADPH/NADP^+^ ratio using the NADPH/NADP^+^ Assay Kit (n = 3/group). Data are presented as mean ± SD. * *p* < 0.05, ** *p* < 0.01.

Using immunofluorescence analysis, we successfully determined the location and subcellular distribution of key enzymes involved in mitochondrial 1C metabolism within the mouse brain (**Supplementary Figure S6A, B**). We focused on the key enzymes involved in mitochondrial 1C metabolism in the mitochondrial matrix because these enzymes are primarily associated with mitochondrial function. To assess their impact on mitochondrial function, we initially examined MMP using TMRE, as our transfected neurons contained EGFP. MMP in neurons was represented by red fluorescence intensity, with CCCP serving as a positive control. The results demonstrated that the knockdown of SHMT2, MTHFD2, or ALDH1L2 significantly reduced red fluorescence intensity under both normal and hemin-treated conditions compared to that in the control group (**Figure 5A, B**). Flow cytometric analysis further confirmed the reduction in MMP upon knockdown of SHMT2, MTHFD2, and ALDH1L2 (**Figure 5C**), confirming the crucial role of SHMT2, MTHFD2, and ALDH1L2 in MMP maintenance. Because mitochondria are the primary sites of ATP production, cellular ATP levels can be used as an indicator of mitochondrial function. Following the knockdown of SHMT2, MTHFD2, and ALDH1L2, a significant decrease in ATP levels was observed, strongly suggesting impaired mitochondrial function (**Supplementary Figure S6C**). Confocal microscopy images stained with MitoTracker revealed extensive mitochondrial fragmentation in neurons after hemin treatment (**Figure 5D**). To investigate whether SHMT2, MTHFD2, and ALDH1L2 influence mitochondrial morphology, we examined the expression of mitochondrial fission proteins (FIS1 and DRP1) and fusion proteins (MFN1 and MFN2). Knockdown of SHMT2, MTHFD2, and ALDH1L2 led to a slight decrease in total DRP1 protein levels and an increase in phosphorylated DRP1 at Ser616, indicating DRP1 activation (**Figure 5E, Supplementary Figure S6D, E**). Additionally, there was an increase in FIS1 expression but a reduction in MFN1 and MFN2 expression (**Figure 5E, Supplementary Figure S6D, E**). These results collectively indicate that hemin stimulation disrupts neuronal MMP and induces a shift in mitochondrial morphology from fusion to fission. In summary, SHMT2, MTHFD2, and ALDH1L2 are essential for maintaining neuronal MMP and regulating mitochondrial shape.

Next, we investigated whether SHMT2, MTHFD2, and ALDH1L2 affected mitochondrial ROS (mROS) levels. Our findings indicated that mROS levels in SHMT2, MTHFD2, and ALDH1L2 knockdown neurons were remarkably higher than those in control neurons (**Figure 5F**). NADPH, an essential byproduct of mitochondrial 1C metabolism, is crucial for cell redox regulation. Our results revealed a significant decrease in the NADPH/NADP^+^ ratio after hemin treatment compared to the control group (**Figure 5G**). Under normal conditions, MTHFD2 or ALDH1L2 knockdown significantly decreased the NADPH/NADP^+^ ratios compared to the control, whereas SHMT2 knockdown also decreased the NADPH/NADP^+^ ratio but not significantly (**Figure 5G**). Following treatment with hemin, the knockdown of SHMT2, MTHFD2, or ALDH1L2 exacerbated the decline in the NADPH/NADP^+^ ratio (**Figure 5G**).

### Key enzymes of mitochondrial 1C metabolism necessary for neuronal survival and induction of ERS following MTHFD2 knockdown

**Figure 6:**
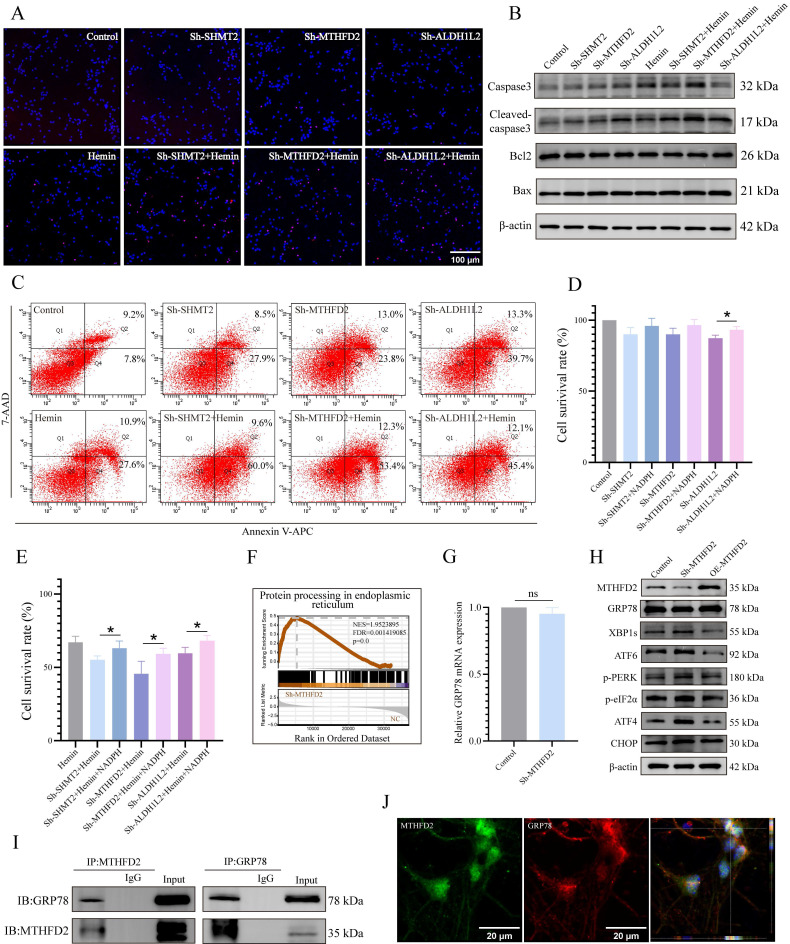
** SHMT2, MTHFD2, and ALDH1L2 are necessary for neuronal survival, and MTHFD2 depletion induces the activation of ERS. A)** Representative confocal images of primary neurons with TUNEL staining in control, hemin-treated groups, and SHMT2, MTHFD2, or ALDH1L2 knocked-down groups. **B)** Representative western blotting results of apoptosis markers in control, hemin-treated groups, and SHMT2, MTHFD2, and ALDH1L2 knockdown groups. **C)** Apoptosis detected using flow cytometry through Annexin V-APC/7-AAD double staining. **D)** Cell viability of SHMT2, MTHFD2, or ALDH1L2 knocked-down neurons measured by CCK-8 assay (n = 3/group). **E)** Cell viability of neurons treated with or without exogenous NADPH in the control, hemin-treated groups, and SHMT2, MTHFD2, or ALDH1L2 knocked-down groups (n = 3/group). **F)** Enrichment score plots from GSEA using RNA sequencing data of MTHFD2 knockdown primary neurons. A list of "protein processing in endoplasmic reticulum" genes was used as the gene set of interest. **G)** The mRNA level of GRP78 was detected using qRT-PCR. **H)** Western blot analysis of ERS marker protein levels in primary neurons with MTHFD2 knockdown or overexpression (n = 3/group). **I)** Co-immunoprecipitation (Co-IP) assay demonstrating the interaction between GRP78 and MTHFD2 in primary neurons. **J)** Confocal microscopy demonstrating the colocalization of GRP78 and MTHFD2 in primary neurons. Data are presented as mean ± SD. * *p* < 0.05, ** *p* < 0.01.

Considering the regulatory role of 1C metabolism genes in mitochondrial function, we further investigated the effects of SHMT2, MTHFD2, and ALDH1L2 on neuronal apoptosis and observed significantly increased TUNEL-positive neurons with silenced SHMT2, MTHFD2, or ALDH1L2 (**Figure 6A, Supplementary Figure S7A**). Subsequently, we examined the influence of 1C metabolism genes on apoptosis using western blotting and flow cytometry (**Figure 6B, C, Supplementary Figure S7B**). The viability of SHMT2, MTHFD2, or ALDH1L2 knockdown neurons also exhibited a downward trend (**Figure 6D**). As previously mentioned, the knockdown of SHMT2, MTHFD2, or ALDH1L2 significantly affects NADPH production. Therefore, we investigated whether exogenous NADPH treatment could reverse neuronal apoptosis. Following supplementation with exogenous NADPH, the viability of neurons in the SHMT2, MTHFD2, and ALDH1L2 knockdown groups increased significantly (**Figure 6E, Supplementary Figure S7C**). These results collectively indicated that exogenous NADPH could mitigate apoptosis in hemin-treated neurons or in those with SHMT2, MTHFD2, or ALDH1L2 knockdown.

To further explore the potential molecular mechanisms underlying the roles of SHMT2, MTHFD2, and ALDH1L2 in maintaining neuronal redox homeostasis and cell survival, we knocked down these genes and performed RNA sequencing. The results revealed a significant enrichment of the "protein processing in endoplasmic reticulum" gene set in MTHFD2 knockdown neurons (**Figure 6F, Supplementary Figure S7E**), leading us to speculate that MTHFD2 might influence ERS. Following MTHFD2 knockdown in primary neurons, we detected ERS activation by western blotting (**Figure 6G, H, Supplementary Figure S7D**). However, unlike the common increase in GRP78 expression observed during ERS, GRP78 protein levels were downregulated after MTHFD2 knockdown, and there was no significant change in GRP78 mRNA levels (**Figure 6G, H**). GRP78 is a vital chaperone involved in maintaining ER protein homeostasis and serves as a primary upstream regulator of ERS. These findings highlighted the regulation of GRP78 expression by MTHFD2 after transcription.

Investigation of the molecular mechanisms associated with the actions of MTHFD2 and GRP78 via a co-IP assay indicated that MTHFD2 and GRP78 were co-localized (**Figure 6I, J**). These data collectively suggest that MTHFD2 interacts with GRP78 to regulate ER homeostasis and that a negative regulatory loop exists between the GRP78/PERK/ATF4 axis and MTHFD2, contributing to maintaining neuronal homeostasis and survival.

### PERK blockade and NADPH supplementation ameliorate neurological function and apoptosis in mice

**Figure 7:**
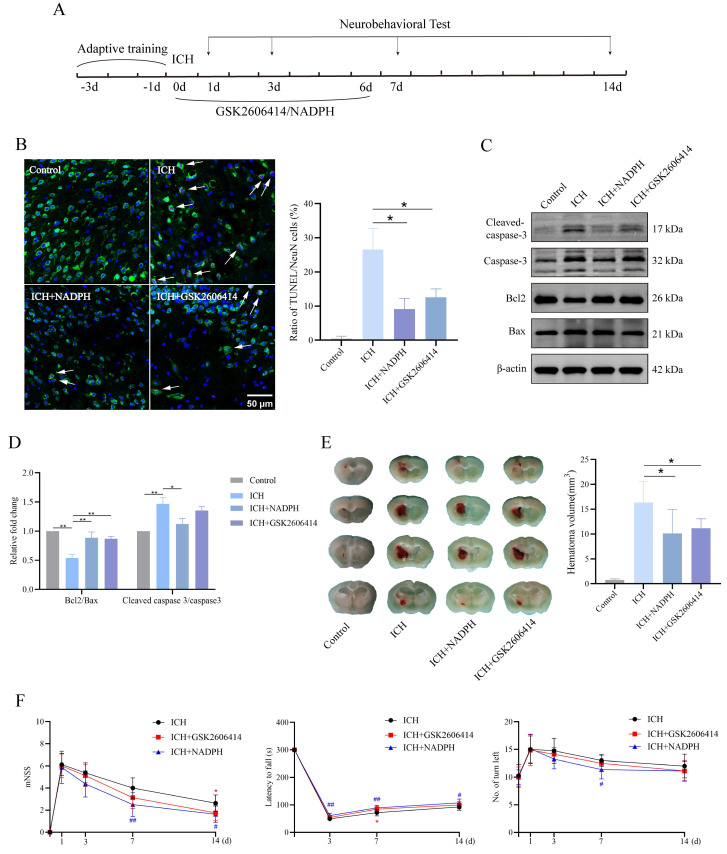
** PERK blockade and exogenous NADPH supplement ameliorate the neurological function and neuronal apoptosis in mice. A)** Schematic overview of the experimental design. **B)** Representative confocal images showing co-staining of TUNEL (red) and NeuN (green) in groups of mice treated with or without GSK2606414 and NADPH (n = 3/group). **C-D)** Western blot analysis of apoptosis markers in protein extracted from perihematomal brain tissues in groups of mice treated with or without GSK2606414 and NADPH after ICH (n = 3/group). **E)** The hematoma volume in ICH mice of different treated groups 3 days after ICH (n = 6/group). **F)** mNSS, rotarod test and EBST test in the four groups of mice (n = 8/group). Data are presented as mean ± SD. * *p* < 0.05, ** *p* < 0.01, ^#^ *p* < 0.05, ^##^ *p* < 0.01.

Here, we investigated whether inhibiting PERK and the 1C metabolic product NADPH mitigates the neurotoxic effects induced by ICH in mice. The experimental timeline is shown in **Figure 7A**. Immunofluorescence and western blot analyses revealed reduced TUNEL signals in the ICH group treated with GSK2606414 and the ICH group supplemented with NADPH, compared to the untreated ICH group (**Figure 7B-D**). Furthermore, brain sections stained with Nissl were examined to assess gross pathological changes among the groups. We found that GSK2606414 and NADPH partially reduced the ICH volume (**Figure 7E**). All mice were subjected to neurobehavioral assessments and received adaptive training for three days prior to ICH induction. Additionally, we evaluated the effects of GSK2606414 and NADPH on the recovery of motor function and observed that the ICH group treated with GSK2606414 and the ICH group treated with NADPH exhibited improved performance in the mNSS, rotarod test, and EBST on day 3 post-ICH compared to the ICH group without treatment (**Figure 7F**). These findings indicate that suppressing the PERK/ATF4 signaling pathway and adding the exogenous 1C metabolic product, NADPH can mitigate ICH-induced neurotoxicity in mice. As mentioned previously, the PERK/ATF4 signaling pathway can activate pro-survival and pro-apoptotic signals under different stress conditions. The reason why inhibition of the PERK/ATF4 signaling pathway reduces neurotoxicity in mice after ICH might be attributed to the fact that sustained harmful stimulation following ICH activates the pro-apoptotic signal mediated by the PERK/ATF4 pathway.

## Discussion

ICH can be a fatal cerebrovascular condition associated with severe cognitive and sensorimotor deficits 3, 37, 38. Existing evidence confirmed the importance of oxidative stress for neuronal damage following ICH 39, 40. In this study, we observed the disruption of 1C metabolism and ERS in neurons following ICH. NADPH, generated through mitochondrial 1C metabolism, is crucial for the cellular redox balance.

**Figure 8:**
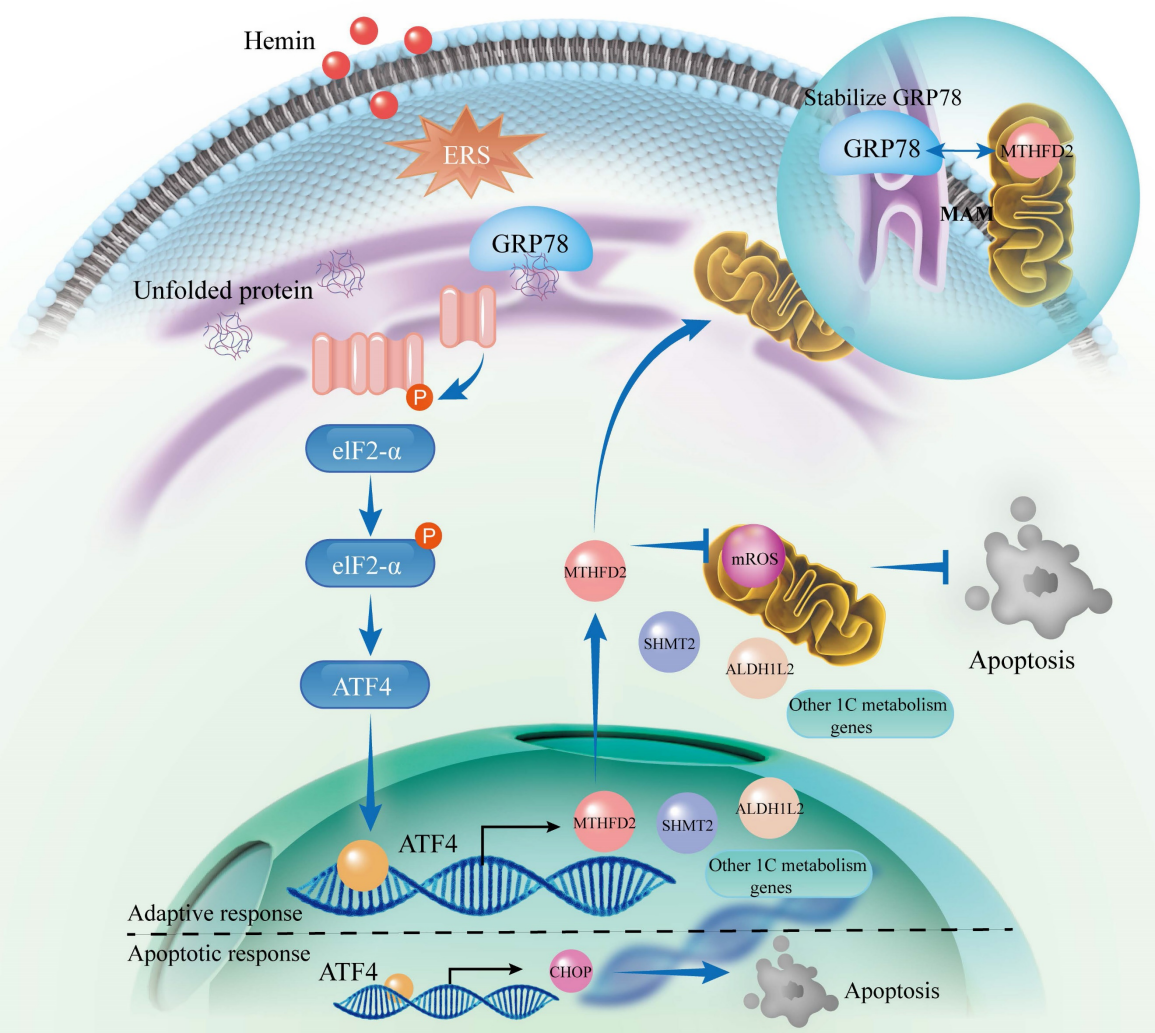
** Schematic illustration of the interaction between the PERK/ATF4 branch of endoplasmic reticulum stress response and mitochondrial one-carbon metabolism after ICH.** ICH causes the accumulation of toxic byproducts in neurons, resulting in the accumulation of unfolded proteins and the subsequent initiation of the ERS. In response to these stresses, an adaptive response occurs through the PERK/ATF4 signaling pathway by increasing 1C metabolism to maintain cellular homeostasis. However, if the adaptive response is insufficient to alleviate ERS, ATF4 activates the CHOP apoptotic pathway. Furthermore, MTHFD2 may play a role in stabilizing GRP78 within the ER, forming a negative regulatory loop with the GRP78/PERK/ATF4 axis to maintain ER homeostasis. After ICH, interventions such as PERK blockade and exogenous NADPH supplementation may mitigate neuronal apoptosis.

Inhibition of ERS by GSK2606414 and supplementation with exogenous NADPH effectively ameliorated neurological function and mitigated neuronal apoptosis in mice. Mechanistically, we identified ATF4 as a key upstream transcription factor that regulates 1C metabolism genes and governs adaptive and apoptotic responses. Under normal physiological conditions, ATF4 regulates various genes, including those involved in 1C metabolism, to maintain cellular homeostasis and represents an adaptive response. Conversely, inadequate adaptive responses during ERS may lead ATF4 to activate the CHOP apoptotic pathway. Additionally, we uncovered the molecular mechanisms underlying the formation of a negative regulatory loop involving the GRP78/PERK/ATF4 axis and MTHFD2 (**Figure 8**).

Brain injuries can occur secondary to ICH, including increased edema, buildup of toxic byproducts, excitotoxicity, oxidative stress, and inflammation 4, 40, 41. These pathophysiological changes trigger oxidative stress and ERS to activate UPR, leading to a neuroinflammatory response and ERS-related neuronal apoptosis 4, 42. Research has shown that neurons are the central focus of UPR targeting and manifest the swiftest UPR activation in the aftermath of brain trauma 16. Furthermore, mice lacking ATF4 display diminished recovery following spinal cord injuries, highlighting the significant involvement of the PERK/ATF4 pathway in neural injury 43. ATF4 is related to cell survival in oxidative stress, is an important factor for redox regulation in cardiomyocytes, and can enhance GSH biosynthesis 23, 44. In this study, we confirmed that ATF4 functions as an upstream transcription factor that directly regulates the expression of genes associated with 1C metabolism using CUT&Tag analysis and ChIP-seq databases and that knockdown of ATF4 led to decreased cellular viability, consistent with prior findings 23, 24, 45. ATF4 plays a central role in orchestrating cellular responses to stress and regulates numerous target genes associated with diverse rescue pathways that enhance cell survival. However, some target genes, such as CHOP, are also involved in apoptosis 46. The behavior of the UPR pathway appears to depend on the extent of ERS, with low levels of ERS activating pro-survival signals and severe and persistent ERS activating ATF4/CHOP apoptotic signals 46, 47. In line with these reports, our experimental results indicated that neuronal activity decreased after ATF4 knockdown under normal conditions, but apoptosis of hemin-treated neurons was reduced following the use of the PERK inhibitor (GSK2606414) *in vivo* or *in vitro*.

1C metabolism has been shown to be essential in numerous cell functions, encompassing biosynthesis (such as purine and thymine synthesis), maintaining the equilibrium of amino acids (i.e., glycine, serine, and methionine), the preservation of epigenetic traits, and protection against oxidative stress 48, 49. The cellular redox state depends on the dynamic balance between ROS generation and activation of antioxidant mechanisms 50. Previous research has demonstrated that reducing key enzymes like SHMT2 or MTHFD2 can decrease the NADPH/NADP^+^ and GSH/GSSG ratios, increase cellular ROS levels, and induce cell death under stress conditions 13, 51. Here, we demonstrate that 1C metabolism is essential for NADPH production and repressing ROS production in the mitochondria. In previous studies, NADPH was reported to be mainly associated with pathways such as PPP, malic enzymes, and isocitrate dehydrogenase, whereas mitochondrial 1C metabolism has primarily been recognized for its role in generating 1C units and nucleotides, without being considered a major source of NADPH 12, 52, 53. However, our findings challenge this notion because we observed that silencing key enzymes involved in mitochondrial 1C metabolism (i.e., SHMT2, MTHFD2, and ALDH1L2) resulted in impaired mitochondrial function and a significant reduction in NADPH production, highlighting the pivotal role of mitochondrial 1C metabolism in maintaining normal mitochondrial function and cellular redox balance. The exact reasons for this metabolic discrepancy, whether influenced by the cell type or other factors, warrant further investigation. Furthermore, our study demonstrated that supplementation with exogenous NADPH could reduce neuronal apoptosis in primary neurons with knockdown of SHMT2, MTHFD2, or ALDH1L2 *in vitro*, which aligns with our *in vivo* results, where exogenous NADPH supplementation reduced ICH volume in mice and improved neurological function scores, possibly because NADPH enhances mitochondrial function and cellular antioxidant defenses.

To determine the molecular mechanisms through which mitochondrial 1C metabolism genes affect neuronal survival, we conducted RNA sequencing after knocking down these metabolic genes (SHMT2, MTHFD2, and ALDH1L2) and observed that MTHFD2 knockdown activated all three branches of the ERS. However, we observed an unexpected phenomenon: the protein expression of GRP78, a key ER chaperone and a central regulator of the ERS, decreased despite ERS activation. GRP78 typically acts as a central regulator by binding to and inhibiting the three ERS sensors (PERK/ATF4, IRE1α/XBP1s, and ATF6) under normal cellular conditions. It is released only from these sensors when misfolded proteins accumulate, thereby triggering the ERS 54. Given this inconsistency, we hypothesized that MTHFD2 regulates GRP78 at the post-transcriptional level, possibly contributing to the stabilization of GRP78 in the ER. Our observations were supported by subsequent CoIP experiments and immunofluorescence assays, which confirmed the binding interaction between MTHFD2 and GRP78. However, the exact mechanism through which MTHFD2 influences GRP78 stability requires further investigation. Previous studies have indicated that MTHFD2 can specifically regulate the PERK/ATF4 axis in glioblastoma cells, and the variation in our findings may be attributed to the differences in cell types and experimental conditions 55. Although MTHFD2 is predominantly localized within mitochondria, it can affect ER homeostasis. Importantly, the dynamic interface known as mitochondria-associated membranes (MAMs) facilitates close communication between the ER and mitochondria, allowing these organelles to execute independent functions while coordinating their responses 56-58. MAMs are pivotal for integrating diverse ER and mitochondrial functions, particularly for transmitting apoptotic signals. The ER and mitochondria can exchange apoptotic signals, exemplifying the synergistic and complementary actions of various organelles within cells 59. Considering that MTHFD2 primarily resides in the mitochondria and that GRP78 predominantly orchestrates the UPR within the ER, the interaction between these two proteins may occur in MAMs. Nevertheless, the present study has not revealed whether MTHFD2 interacts directly with GRP78 or employs intermediary proteins in this process, a topic that merits additional in-depth exploration.

In summary, our findings elucidate a synergistic mechanism involving PERK/ATF4 signaling triggered by ERS and mitochondrial one-carbon metabolism in preserving neuronal homeostasis following ICH. We also demonstrated the existence of a negative regulatory loop between the GRP78/PERK/ATF4 axis and MTHFD2. The neuroprotective effects of mitochondrial 1C metabolism after ICH were found to be associated with NADPH production and maintenance of ER homeostasis. Interventions using exogenous NADPH supplementation and modulating the PERK/ATF4 axis hold promise for alleviating neuronal damage symptoms and may contribute to identifying potential targets for therapeutic application.

## Supplementary Material

Supplementary figures and tables.

## Abbreviations

ICH: intracerebral hemorrhage
ER: endoplasmic reticulum
ERS: endoplasmic reticulum stress
1C: one-carbon
G6PD: glucose-6-phosphate dehydrogenase
UPR: unfolded protein response
ROS: reactive oxygen species
PFA: paraformaldehyde
IF: immunofluorescence
PBS: phosphate-buffered saline
FC: fold change
MMP: mitochondrial membrane potential
RTP: room temperature
UPLC-MS/MS: ultra-high-performance liquid chromatography-tandem mass spectrometry
PCA: principal component analysis
PPP: pentose phosphate pathway
OPLS-DA: orthogonal projections to latent structures-discriminant analysis
NSS: neurological severity score
EBST: elevated body swing test
