# Supplementary material for: Interaction Between the PERK/ATF4 Branch of the Endoplasmic Reticulum Stress and Mitochondrial One-Carbon Metabolism Regulates Neuronal Survival After Intracerebral Hemorrhage
Source: Int J Biol Sci. 2024 Aug 6;20(11):4277–96. doi: 10.7150/ijbs.93787 (PMC11379068; doi:10.7150/ijbs.93787)
Supplement: Supplementary file 1 — Supplementary figures and tables. [file ijbsv20p4277s1.pdf]

**Supplementary figures**

**Figure S1**

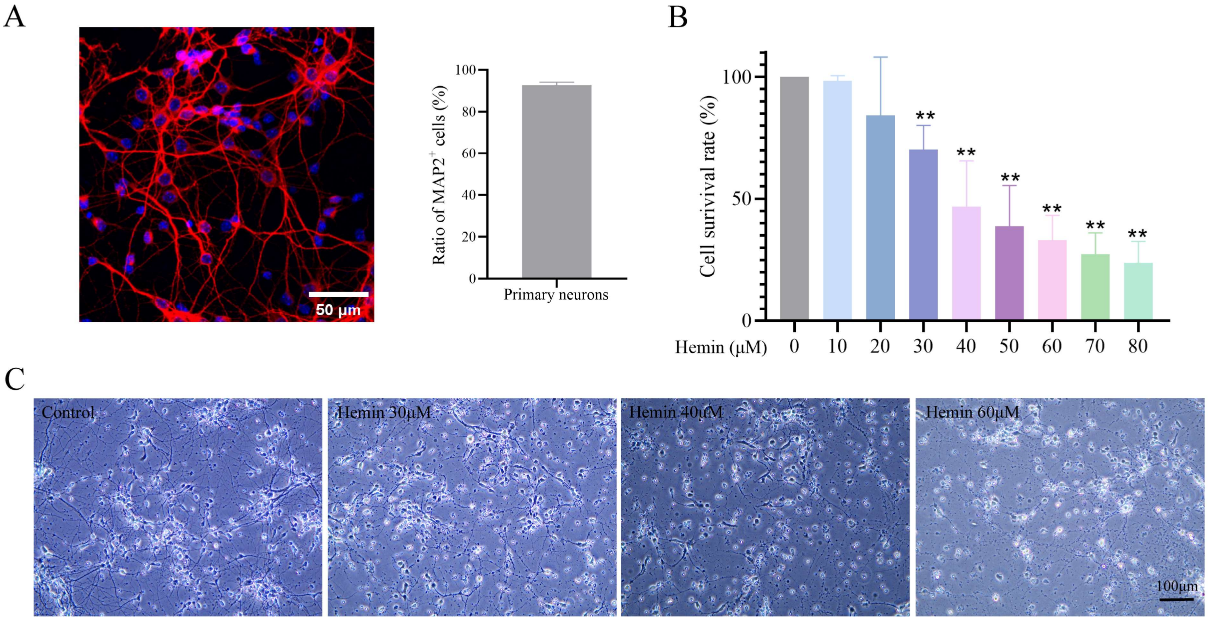


**Figure S1. A)** MAP2 expression of primary neurons, revealing a purity level of at least greater than 90%. **B)** CCK-8 assay detecting the cell survival rates of neurons 24 h after stimulation with different concentrations of hemin (n=3/group). **C)** Typical morphological characteristics of primary neurons after being treated with the indicated hemin concentrations for 24 h. Data are presented as mean ± SD. ** $p < 0.01$.

**Figure S2**

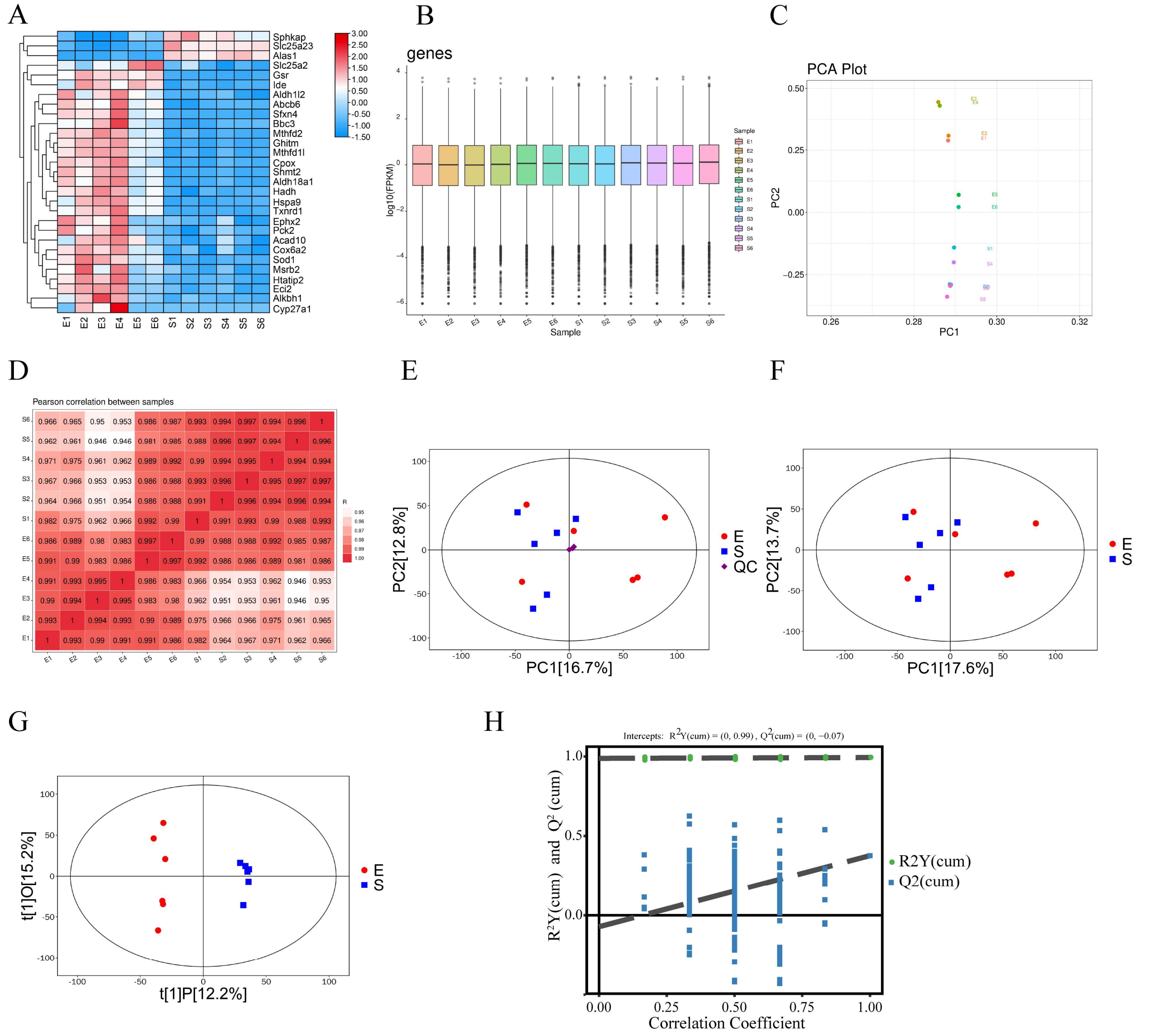


**Figure S2. A)** Heatmap displaying the expression levels of 29 differentially expressed genes that overlap with MitoCarta3.0, based on data from n=6 per group. (S represents control neurons; E represents hemin-treated 24-h neurons). **B)** Box plot depicting the FPKM values of RNA sequencing samples, demonstrating the reproducibility of the samples. **C–D)** PCA plot and Pearson correlation coefficient between samples showing good repeatability on RNA sequencing samples. **E)** Score scatter plot of PCA for all samples, including quality control samples, in non-targeted metabolomics analysis. **F)** Score scatter plot of the PCA model for two groups, showing that all samples are within the 95% confidence interval. **G)** Score scatter plot of the OPLS-DA model for two groups, indicating excellent reproducibility within each group. **H)** Permutation plot test of the OPLS-DA model for two groups, confirming the model's reliability.

## Figure S3

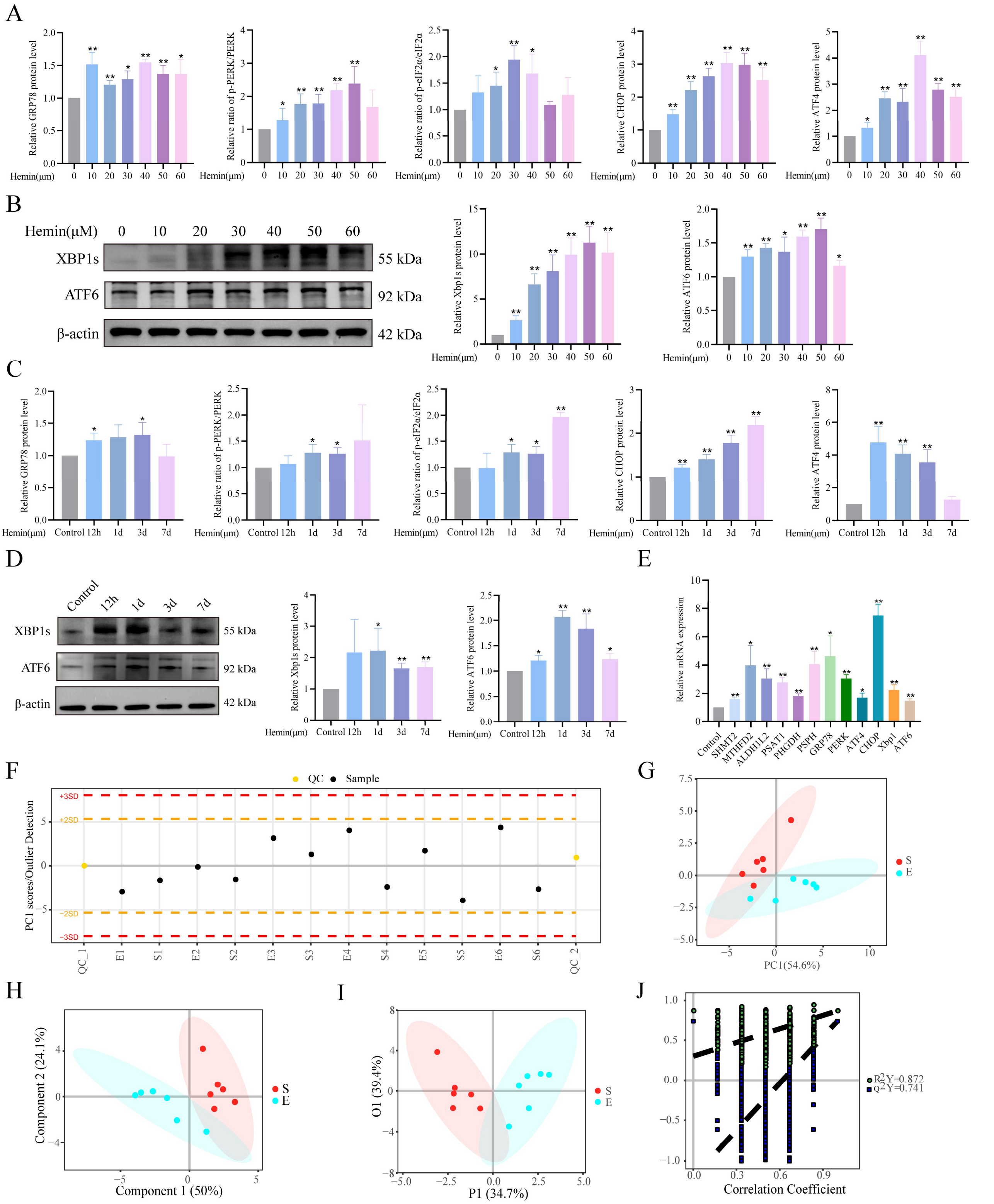


**Figure S3. A)** Expression levels of GRP78, CHOP, ATF4, the p-PERK/PERK, and p-eIF2α/eIF2α protein ratio after stimulation with different concentrations of hemin (n = 3/group). **B)** Western blot analysis showing the levels of ATF6 and IRE1α/XBP1s branch markers in the ERS after primary neurons were treated with

varying concentrations of hemin for 24 h (n = 3/group). **C)** Expression levels of GRP78, CHOP, ATF4, the p-PERK/PERK, and p-eIF2α/eIF2α protein ratio in protein extracts from perihematomal mouse brain tissue at different time points after ICH (n = 3/group). **D)** Western blot analysis of ATF6 and IRE1α/XBP1s branch markers in the ERS in protein extracts from perihematomal mouse brain tissue at different time points after ICH (n = 3/group). **E)** qRT-PCR analysis of key genes involved in the ERS and 1C metabolism in RNA extracted from primary neurons treated with or without 30 μM hemin for 24 h (n = 3/group). **F)** Multivariate control chart illustrating targeted metabolomics data for 1C-related substances, where each point represents a sample. All samples remain within 2 standard deviations, indicating good quality control and reliable data. **G)** Two-dimensional score plot of PCA for the two groups, visually representing patterns between different groups. **H)** Two-dimensional score plot of PLS-DA for the two groups. **I)** Two-dimensional score plot of OPLS-DA for the two groups for selecting differential metabolites. **J)** Permutation test results for the OPLS-DA model for the two groups to assess the model's validity (n=6 per group. S represents control neurons; E represents hemin-treated 24-h neurons). Data are presented as mean ± SD. * $p < 0.05$, ** $p < 0.01$.

## Figure S4

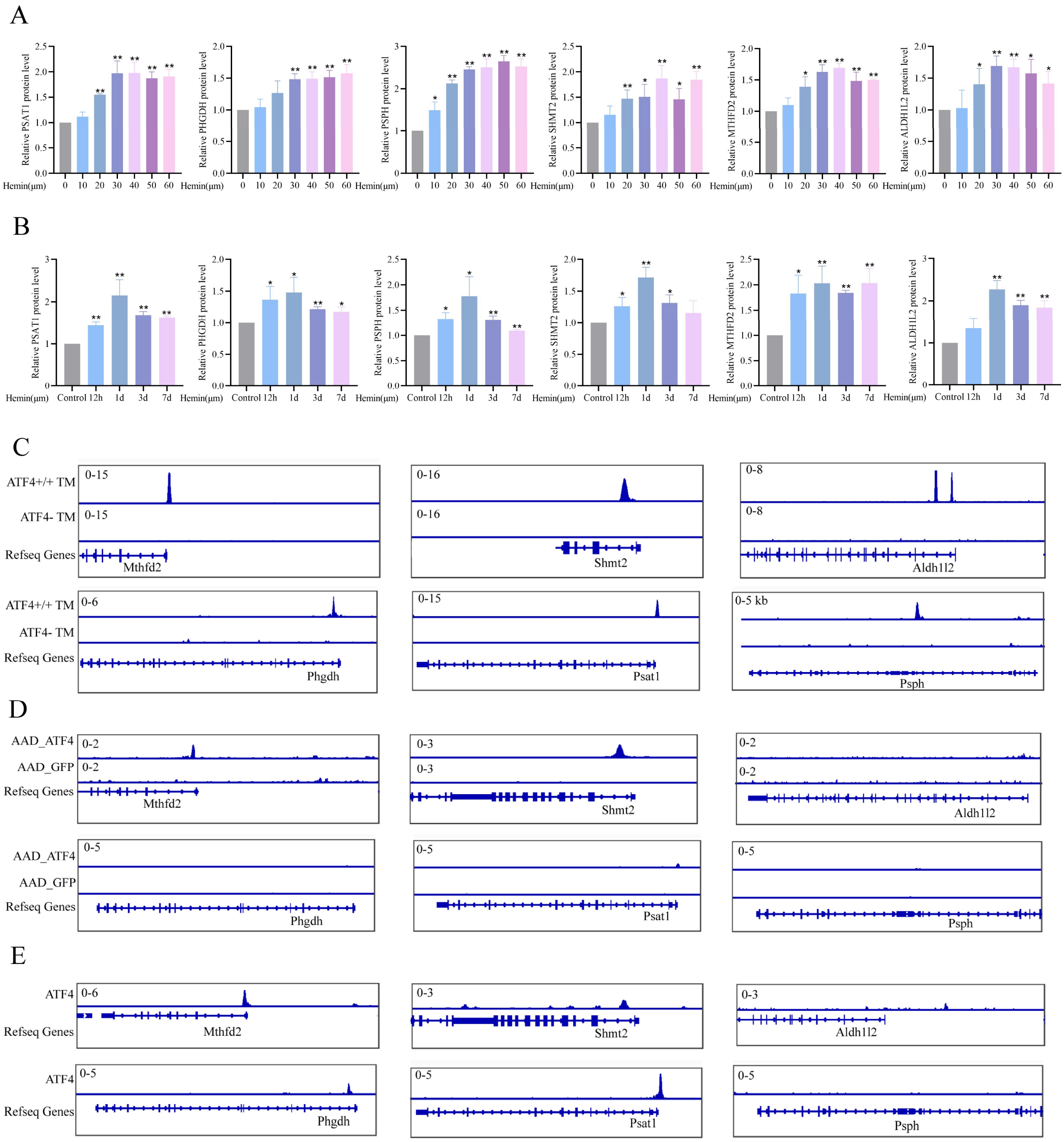


**Figure S4. A)** Expression levels of PSAT1, PHGDH, PSPH, SHMT2, MTHFD2, and ALDH1L2 after stimulation with different concentrations of hemin (n = 3/group). **B)** Expression levels of PSAT1, PHGDH, PSPH, SHMT2, MTHFD2, and ALDH1L2 in protein extracts from perihematomal mouse brain tissue at different time points after ICH (n = 3/group). **C–E)** Chromatin Immunoprecipitation-Sequencing (ChIP-seq) databases were utilized to visualize genome browser views of ATF4-binding peaks in proximity to transcription start sites of SHMT2, MTHFD2, ALDH1L2, PHGDH, PSAT1, and PSPH genes (GSE35681, GSE75165, and GSE44338). Data are presented as mean ± SD. * $p < 0.05$, ** $p < 0.01$.

**Figure S5**

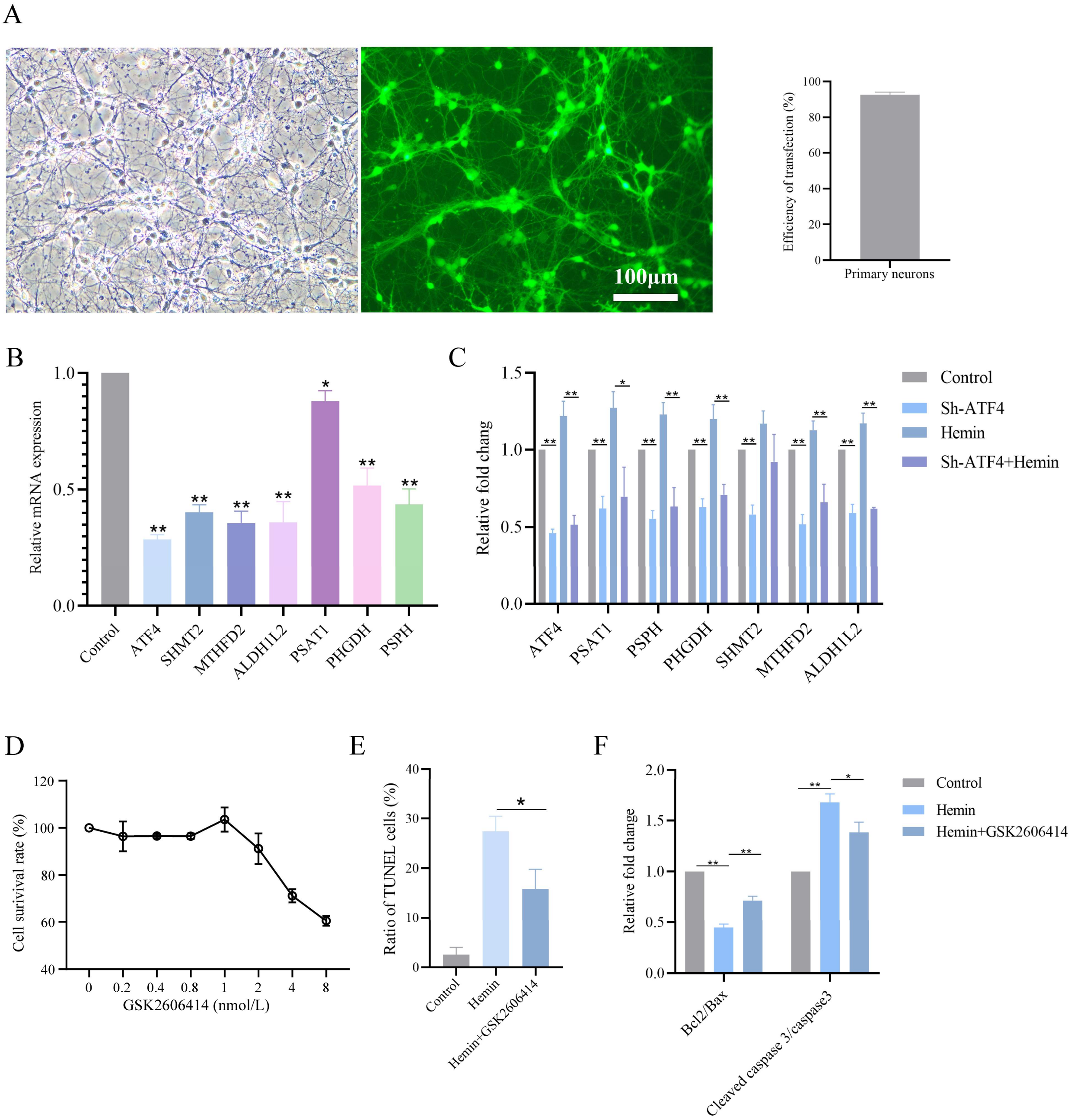


**Figure S5. A)** Transfection efficiency of the lentiviral vector approached 90% when the multiplicity of infection (MOI) was 10 in primary neurons. **B)** Detection of expression levels of 1C metabolism genes by qRT-PCR in RNA extracted from primary neurons following ATF4 knockdown (n = 3/group). **C)** Protein expression levels of ATF4, PSAT1, PHGDH, PSPH, SHMT2, MTHFD2, and ALDH1L2 in primary neurons after ATF4 knockdown, assessed by WB analysis (n = 3/group). **D)** Neuronal activity following treatment with varying concentrations of GSK2606414 for 24 h using the CCK-8 kit (n = 3/group). **E)** Quantitative analysis of TUNEL+ apoptotic primary neurons treated with or without GSK2606414 for 24 h (n =3/group).

**F)** Bcl2/Bax and cleaved caspase-3/caspase-3 expression levels in neurons treated with or without GSK2606414 under hemin stimulation conditions, analyzed by western blot analysis (n = 3/group). Data are presented as mean ± SD. * $p < 0.05$, ** $p < 0.01$.

**Figure S6**

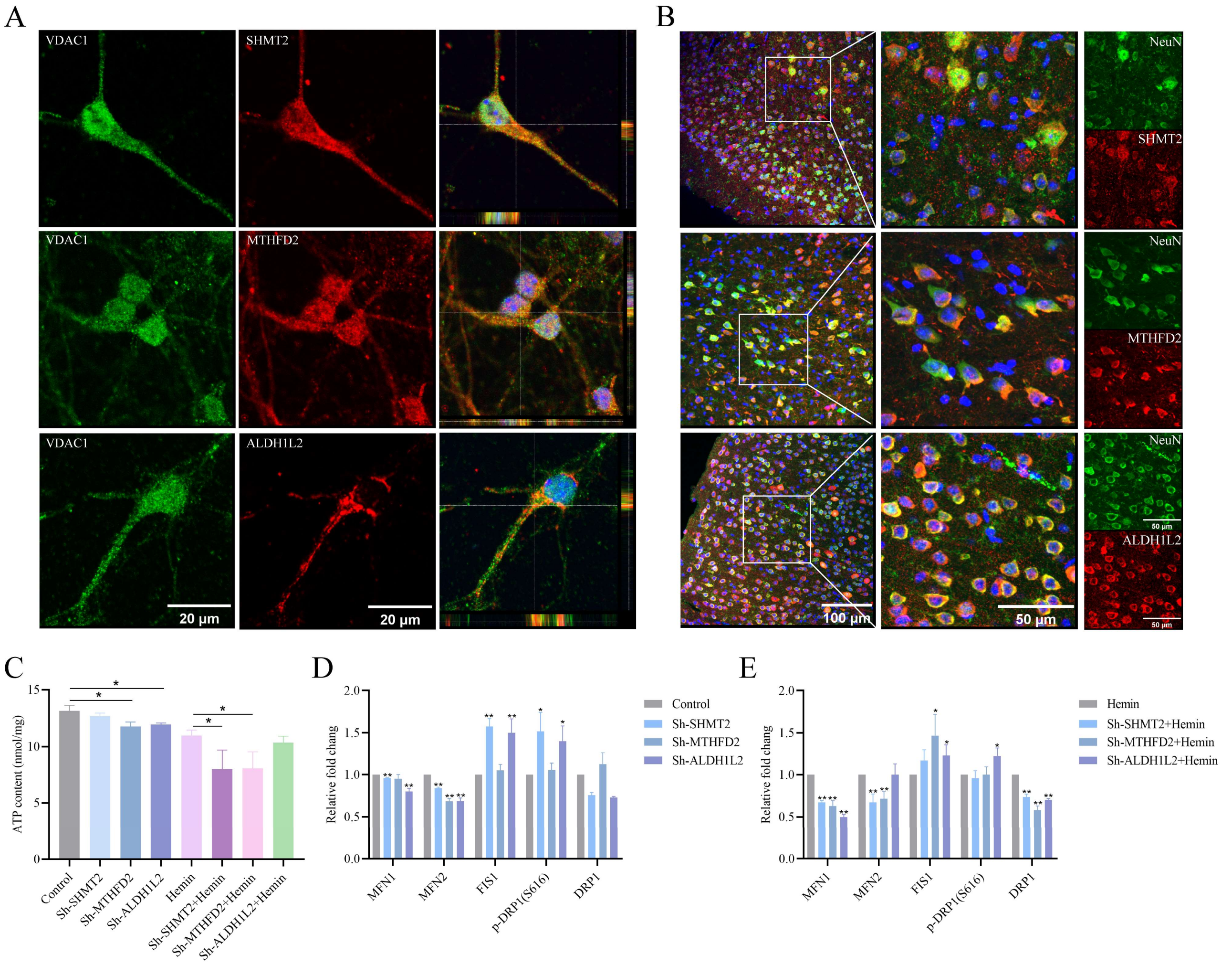


**Figure S6. A)** Confocal microscopy demonstrating the colocalization of SHMT2, MTHFD2, and ALDH1L2 with a mitochondrial marker (VDAC1) in primary neurons. **B)** Confocal microscopy illustrating the colocalization of SHMT2, MTHFD2, and ALDH1L2 with a neuron marker (NeuN). **C)** Measurement of cellular ATP content using the ATP Assay Kit (n = 3/group). **D)** Analysis of the expression levels of MFN1, MFN2, FIS1, p-DRP1(S616), and DRP1 after knockdown of the key enzymes of mitochondrial 1C metabolism (SHMT2, MTHFD2, and ALDH1L2) (n = 3/group). **E)** Analysis of the expression levels of MFN1, MFN2, FIS1, p-DRP1(S616), and DRP1 after stimulation with hemin and knockdown of the key enzymes of mitochondrial 1C metabolism (SHMT2, MTHFD2, and ALDH1L2) (n = 3/group). Data are presented as mean ± SD. * $p < 0.05$, ** $p < 0.01$.

**Figure S7**

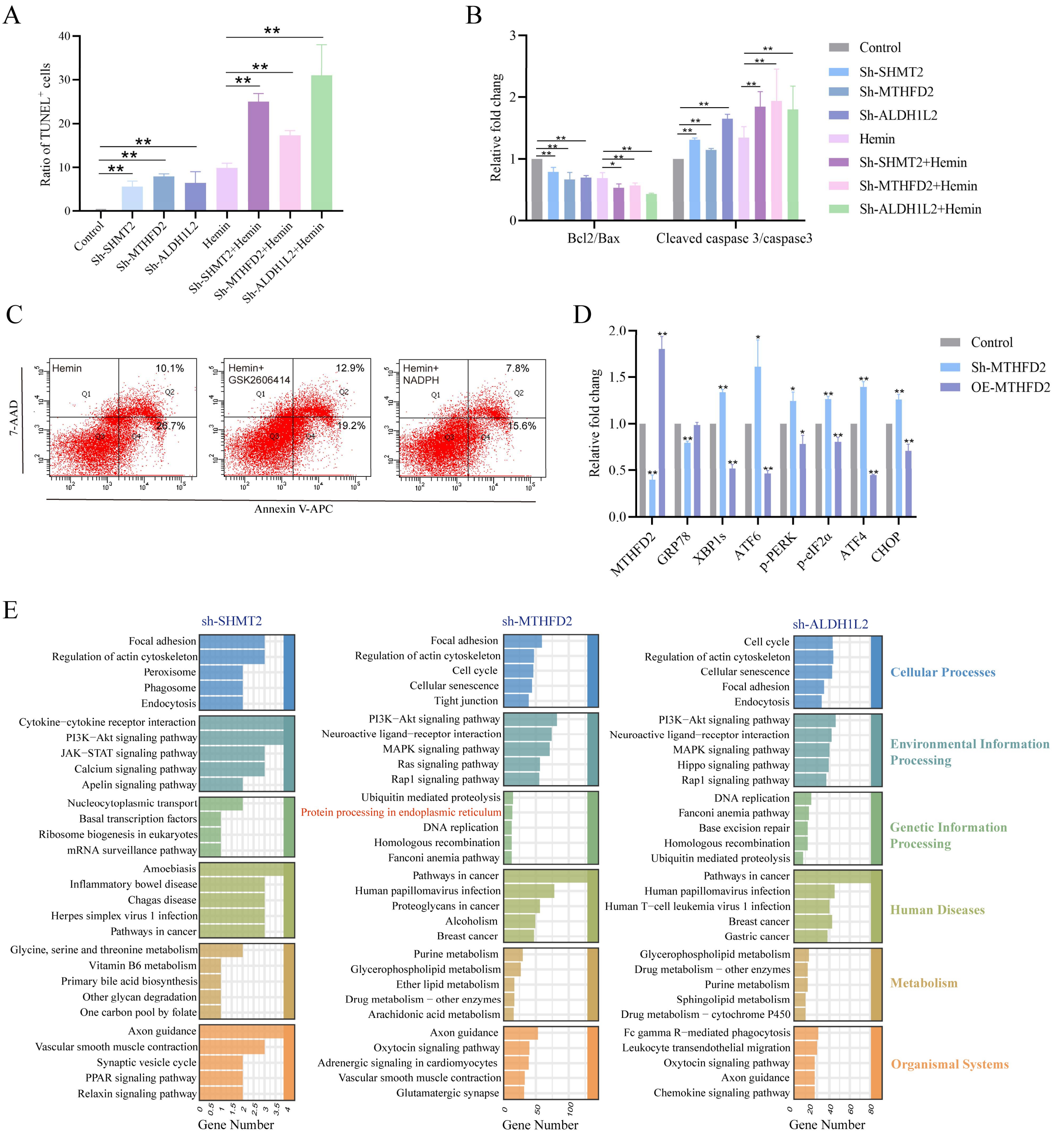


**Figure S7. A)** Quantitative analysis of TUNEL+ apoptotic primary neurons following knockdown of the key enzymes of mitochondrial 1C metabolism (SHMT2, MTHFD2, and ALDH1L2) (n = 3/group). **B)** Western blot analysis showing changes in Bcl2/Bax and cleaved caspase-3/caspase-3 expression after knockdown of the key enzymes of mitochondrial 1C metabolism (SHMT2, MTHFD2, and ALDH1L2) in primary neurons (n = 3/group). **C)** Neurons were double-stained with Annexin V-APC/7-AAD, and apoptosis was detected by flow cytometry under hemin stimulation conditions in neurons treated with or without GSK2606414 and

NADPH. **D)** Western blot analysis assessing the levels of ERS marker proteins in primary neurons with MTHFD2 knockdown or overexpression (n = 3/group). **E)** KEGG pathway classification of differentially expressed genes compared with the control group after knockdown of SHMT2, MTHFD2, or ALDH1L2. Data are presented as mean ± SD. * $p < 0.05$, ** $p < 0.01$.

**Supplementary tables**

**Table S1. Primer sequences used for qRT-PCR.**

| Name | Forward (5'-3') | Reverse (5'-3') |
|---|---|---|
| GAPDH | AAATGGTGAAGGTCGGTGTG | AGGTCAATGAAGGGGTCGTT |
| SHMT2 | CTGCAGAGGGAGAAGGACAG | CTCGGCTGCAGAAGTTCTCT |
| MTHFD2 | CCTTGTTGTCTGCGTTGGCT | ATGACAACGGCTTCATTTCGCA |
| ALDH1L2 | ACCAGCCGGGTTTATTTCAAA | ACTCCCACTACTCGGTGGC |
| PSAT1 | TACTTCTGTGCAAACGAGACTG | CACCAGCGAAAATCACACCA |
| PHGDH | ATGGCCTTCGCAAATCTGC | AGTTCAGCTATCAGCTCCTCC |
| PSPH | AGGAAGCTCTTCTGTTCAGCG | GAGCCTCTGGACTTGATCCC |
| GRP78 | CCGAGGAGGAGGACAAGAAGGAG | GAACACACCGACGCAGGAATAGG |
| PERK | GGACGAATCGCTGCACTGGATG | GGCTTGCTGAGGCTAGATGAAACC |
| ATF4 | TCTGCCTTCTCCAGGTGGTTCC | GCTGCTGTCTTGTTTTGCTCCATC |
| CHOP | CCCTCGCTCTCCAGATTCCAGTC | TCGTTCTCCTGCTCCTTCTCCTTC |
| XBP1 | TTGGGCATTCTGGACAAGTTGGAC | ACAGAGAAAGGGAGGCTGGTAAGG |
| ATF6 | TCGCCTTTTAGTCCGGTTCTT | GGCTCCATAGGTCTGACTCC |

**Table S2. Primer sequences used for CHIP-qPCR.**

| Name | Forward (5'-3') | Reverse (5'-3') |
|---|---|---|
| PSAT1 | TCACTGATTGGCTCTCGCTC | TGACCGCTAAAGCCGATTGA |
| PHGDH | GCCAATGGTGGAGGTTGACT | CAAGACCTTGGCGCTCTTTC |
| SHMT2 | AAGTACTGAGGCTCGGTTGC | GGCTTCGATGCGCTAGTTGA |
| MTHFD2 | CAAGGCTAGAACTGGTGGGC | AGGGTACCAACTTCCCTCCT |
| ALDH1L2 | TGTCGGCTCTCGTTCCATTT | GAGTAGTGGCGGCCAGATTT |

**Table S3. shRNA sequences.**

| Name | TargetSeq |
|---|---|
| NC | TTCTCCGAACGTGTCACGT |
| SHMT2 | CTTCGAGTCTATGCCCTATAA |
| MTHFD2 | GCTCATGAAGAACACCATTAT |
| ALDH1L2 | GGTGTTGCAGAGAGCAAATAA |
| ATF4 | GCGAGTGTAAGGAGCTAGAAA |

**Table S4. 1C metabolism-related metabolites detected by targeted metabolomics.**

| HMDB | KEGG | Metabolite |
|---|---|---|
| HMDB0000696 | C00073 | Methionine |
| HMDB0000123 | C00037 | Glycine |
| HMDB0000187 | C00065 | Serine |
| HMDB0000574 | C00097 | Cysteine |
| HMDB0000099 | C02291 | Cystathionine |
| HMDB0000192 | C00491 | Cystine |
| HMDB0001185 | C00019 | SAM |
| HMDB0000939 | C00021 | SAH |
| HMDB0000676 | C01817 | Homocystine |
| HMDB0001396 | C00440 | 5-MTHF |
| HMDB0001562 | C03479 | Folinic acid |
| HMDB0000121 | C00504 | Folic acid |
| HMDB0000902 | C00003 | $NAD^+$ |
| HMDB0000217 | C00006 | $NADP^+$ |

## Supplementary methods

### Method S1

### Non-targeted metabolites extraction and analysis

### Sample preparation

The sample was added with 200 µL of water, swirled for 30 s, and thawed three times with liquid nitrogen. They were then treated with ultrasound in an ice bath for 10 minutes. Homogenate 50 µL for protein quantification. Homogenate 150 µL and add 600 µL(-40°C) of pre-cooled extract containing isotopically labeled internal standard mixture (methanol: acetonitrile = 1:1). Swirl for 30 s, ultrasound in ice water bath for 10 min, incubate at -40°C for 1 h, centrifuge at 12000 rpm at 4°C for 15 min. The resulting supernatant was transferred to a fresh glass vial for LC/MS analysis. Quality control (QC) samples are prepared by mixing the supernatant of all samples in equal parts.

### LC-MS/MS analysis

The Vanquish (Thermo Fisher Scientific) ultra-high performance liquid chromatograph was used in this project. The target compounds were separated by Waters ACQUITY UPLC BEH Amide (2.1 mm x 100 mm, 1.7µm) liquid chromatography column. Phase A was aqueous, containing 25 mmol/L ammonium acetate and 25 mmol/L ammonia hydroxide in water (pH=9.75), and phase B was acetonitrile. The auto-sampler temperature was 4 °C, and the injection volume was 2µL. The Thermo Q Exactive HFX mass spectrometer is capable of primary and secondary mass spectrometry data acquisition under the control of the control software (Xcalibur, Thermo). Detailed parameters are as follows: sheath gas flow rate as 30 Arb, Aux gas flow rate as 25 Arb, capillary temperature 350 °C, full MS resolution as 120000, MS/MS resolution as 7500, collision energy as 10/30/60 in NCE mode, spray Voltage as 3.6 kV (positive) or -3.2 kV (negative), respectively.

### Data processing and analysis

After the original data is converted into mzXML format by ProteoWizard software, the selfcompiled R program package (kernel is XCMS) is used for peak identification, extraction, alignment and integration, and then matched with BiotreeDB (V2.1) self-built secondary mass spectrometry database for material annotation. The cutoff value for the algorithm score is set to 0.3.

**Method S2**

**Targeted metabolites extraction and analysis**

**Sample preparation**

The mass spectrometric pure methanol, acetonitrile and formic acid used in this experiment were purchased from Thermo-Fisher Scientific (FairLawn, NJ, USA). The target substance standard and AQC (6-aminoquinoline-n-hydroxysuccinimate) were purchased from Sigma Corporation (St. Louis, MO, USA). The standard product is dissolved in 50% methanol and prepared into a mother liquor with a concentration of 1.0mg/ml, and then diluted into a series of concentration standard samples to obtain the standard curve.

AQC derivative reagent (1.5mg/ml): Accurately weigh 1.5mg AQC powder into a clean and dry glass vial, add 1000uL dehydrated acetonitrile, and fully dissolve at 55°C (not more than 15min). To reduce degradation, the sample is thawed under an ice bath. Each sample was added with 100μL frozen methanol solution containing inner target, and then centrifuged at 4 °C at a speed of 14000g for 20min after ultrasonic breaking. The 50μL supernatant was transferred to the 96-well plate and affixed with a plastic sealing film for the detection of leaf acids. For amino acids, another 10μL supernatant was taken, and then 70μL boric acid buffer and 20μL AQC derivative reagent were added in turn. After the reaction at 55°C for 10 minutes, 900μL deionized water was added to dilute it. After mixing, take 100μL supernatant and dilute it 10 times before testing on the machine.

**LC-MS/MS analysis**

In this project, ultra-high performance liquid chromatography-tandem mass spectrometry (UPLC-MS/MS) (ACQUITY UPLC-Xevo TQS, Waters Corp., Milford, MA, USA) was used to detect metabolites associated

with carbon metabolism. The parameters and methods of the instrument are set as follows: Column: Folate: ACQUITY UPLC BEH C18 1.7µM analytical column (2.1 × 100 mm) Amino acid: HSS T3 C18 1.8µM analytical column (2.1 × 100 mm); Column temperature (°C) Folate: 40; Amino acid: 45; Mobile phase: A=0.1% formic acid water; B=0.1% acetonitrile formate;

Elution conditions: Folate: 0-2 min (2-5%B), 2-3 min (5-90%B), 3-3.8 min (90%B), 3.8-4 min (90-2%B), 4-5 min (2%B). Amino acids: 0-0.5 min (B) 4%, 0.5 to 2.5 min (0.5 to 2.5% B), 2.5 5 min (B) 10-28%, 5-6 min (B) 28-95%, 6-7 min 95% (B), 7-7.7 min(95-4%B),7.7-9min(4%B); Flow rate (mL/min): Folic acid: 0.4; Amino acid: 0.6; Injection volume (µL): 5.0; Capillary voltage: 3KV (ESI+); Source temperature (°C): 150; Desolvent temperature (°C): 500; Desolvent flow (L/Hr) 1000.

**Data processing and analysis**

MassLynx software (v4.1, Waters, Milford, MA, USA) was used to process the raw data file generated by UPLC-MS/MS to integrate, calibrate, and quantify the peaks of each metabolite. The self-developed iMAP software (V1.0, MetaboProfile, Shanghai, China) was used for subsequent statistical analysis, such as PCA, OPLS-DA and unidimensional statistical test.
